# Downregulation of 4-HNE and FOXO4 collaboratively promotes NSCLC cell migration and tumor growth

**DOI:** 10.1038/s41419-024-06948-4

**Published:** 2024-07-31

**Authors:** Tianfei Zhong, Ying Li, Meng Jin, Jingqun Liu, Zhenyu Wu, Feiye Zhu, Lisha Zhao, Yongsheng Fan, Li Xu, Jinjun Ji

**Affiliations:** 1 College of Basic Medical, Zhejiang Chinese Medical University, Hangzhou, China; 2 Logistic Affairs Department, Zhejiang Chinese Medical University, Hangzhou, China; 3 The First School of Clinical Medicine, Zhejiang Chinese Medical University, Hangzhou, China; 4 Key Laborat Laboratory of Chinese Medicine Rtheumatology of Zhejiang Province, Hangzhou, China; 5 Academy of Chinese Medical Sciences, Zhejiang Chinese Medical University, Hangzhou, China; 6 Department of Medicine, Zhejiang Academy of Traditional Chinese Medicine, Hangzhou, China; 7 Department of Rheumatology, The Second Affiliated Hospital of Zhejiang Chinese Medical University, Hangzhou, China

**Keywords:** Cancer microenvironment, Non-small-cell lung cancer, Non-small-cell lung cancer

## Abstract

Non-small cell lung cancer (NSCLC) is among the most prevalent cancers and a leading cause of cancer-related mortality globally. Extracellular vesicles (EVs) derived from NSCLC play a pivotal role in lung cancer progression. Our findings reveal a direct correlation between the abundance of EVs and the transfection efficiencies. Co-culturing two different lung cancer cell lines could enhance EVs formation, cell proliferation, migration and tumorigenicity. mRNA chip and metabolic analyses revealed significant alterations in the FOXO signaling pathway and unsaturated fatty acid metabolism within tumor tissues derived from co-cultured cells. Shotgun lipidomics studies and bioinformatics analyses guided our attention towards 4-Hydroxynonenal (4-HNE) and FOXO4. Elevating 4-HNE or FOXO4 levels could reduce the formation of EVs and impede cell growth and migration. While silencing FOXO4 expression lead to an increase in cell cloning rate and enhanced migration. These findings suggest that regulating the production of 4-HNE and FOXO4 might provide an effective therapeutic approach for the treatment of NSCLC.

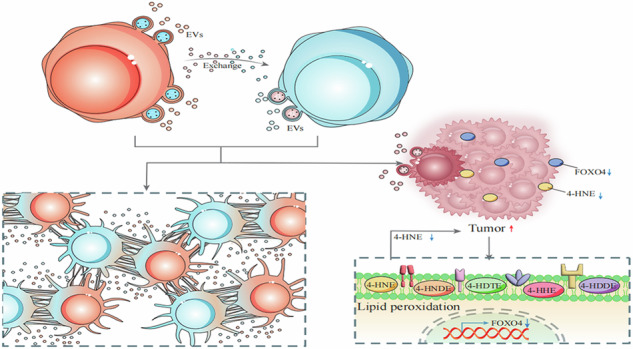

## Introduction

Lung cancer is one of the most frequently cancers leading cause of cancer-related deaths worldwide and non-small cell lung cancer (NSCLC) is a main histological subtype (85% of lung cancer cases) [1]. There is increasing evidence that the tumor microenvironment (TME) is closely related to the development of cancer [2]. The impact of tumor-released extracellular vesicles (EVs) on persistent cellular proliferation and resistance to cell death, stimulation of angiogenesis, facilitation of invasion and metastasis, evasion of immune response, and modification of TME underscores their significance as central regulators in cancer mechanisms [3, 4]. Metastasis of NSCLC is the main cause of death, the specific mechanism of NSCLC metastasis remains to be further studied.

EVs are a heterogeneous population of cell-derived membrane vesicles that are secreted by various cell types [4]. Two main types of EVs are distinguished based on their biogenesis, known as exosomes and ectosomes. Exosomes are small EVs of endosomal origin released by the exocytosis of multivesicular bodies and amphisomes. Ectosomes are formed via the budding and blebbing of the plasma membrane [5, 6]. This process is accompanied by molecular rearrangements at the periphery of the plasma membrane, resulting in modifications to its lipid and protein compositions [4]. EVs released by NSCLC cells can drive invasion and permeability in non-tumorigenic lung epithelial cells [7].

The biogenesis and release of EVs have been shown to increase in various stressful conditions including hypoxia, senescence or oncogene activation [8, 9]. Oxidative stress conditions influence the release and the molecular cargo of EVs that, in turn, modulate the redox status of target cells [9]. To cope with abnormal energy and redox environments, tumor cells upregulate the expression of antioxidant proteins, aiming to detoxify heightened levels of reactive oxygen species (ROS). This process enables the establishment of a redox equilibrium, all the while sustaining pro-tumorigenic signaling and fortifying resistance to apoptosis [10]. Overproduction of these ROS, can disrupt cell signaling, and in turn change the volume and composition of EVs [11]. Accompanied by provoke oxidation of polyunsaturated fatty acids (PUFAs) in cellular membranes through free radical chain reactions and form lipid hydroperoxides as primary products. A study reported that adipocytes respond to oxidative stress by rapidly and robustly releasing EVs [12]. Tumor cells upregulate their lipid metabolism machinery to meet the increased energy demand [13]. Tumor cells regulate intercellular lipid exchange by forming EVs, which may be an effective pathway for them to resist lipid peroxidation death caused by hypoxia-induced oxidative stress.

Lipid peroxidation is a process under which oxidants such as free radicals attack lipids which containing carbon-carbon double bond(s), especially PUFAs, almost all tumor cells have an imbalance in this process [14]. In terms of EVs trafficking, it should be mentioned that the lipid composition of EVs membranes may play a role in conferring vesicles stability or to facilitate uptake into recipient cells [15]. Among lipid peroxidation products, 4-HNE represents one of the most bioactive and well-studied lipid alkenals [16]. A study reported, 4-HNE promotes the release of microvesicles from perivascular cells into the circulation [17]. Prolonged exposure to oxygen fractions exceeding 0.8 (O_2_ fractions) has been shown to induce the production of ROS, leading to the development of pulmonary edema and proliferative fibrosis in animal models [18]. 4-HNE was reported to implicate in this type of acute lung injury. It interacts with various mitochondrial targets, forming adducts that result in mitochondrial dysfunction and disruption of cellular bioenergetics [19]. 4-HNE has a dual and hormetic effect, low concentrations (about 0.1–5 micromolar) are beneficial to cells, promoting proliferation, differentiation, antioxidant defense and compensatory mechanisms, high concentrations (about 10–20 micromolar) trigger toxic pathways leading to cell death. Overproduction of 4-HNE may ultimately compromise membrane integrity and lead to cell death via a process known as ferroptosis [20]. 4-HNE, a marker of lipid peroxidation, is closely associated with lung tumor size and stage. Lung tumors exhibiting higher degree of malignancy tend to show lower level of lipid peroxidation [21]. Inhibiting the excessive production of 4-HNE in tumor tissue maybe is necessary to maintain the survival of tumor cells.

FOXOs are recognized as tumor suppressors due to their confirmed roles in inducing cell cycle arrest, DNA damage repair, and scavenging of ROS [22]. Numerous histopathological investigations have highlighted an association between decreased FOXO4 expression and heightened cancer metastasis [23, 24]. It activates the cell cycle-dependent kinase inhibitor p27, which in turn inhibits cell cycle-dependent kinases (CDKs) and blocks G1 cell cycle progression in tumors [25]. In hypoxic tumor environments, FOXO4 downregulates hypoxia-inducible factor 1α (HIF-1α), thereby suppressing responses to hypoxia such as the expression of glucose transporter type 1 (GLUT-1), erythropoietin (EPO), and vascular endothelial growth factor. These factors are involved in glucose metabolism, erythropoiesis, and angiogenesis, all of which are crucial for tumor development [26]. Furthermore, downregulation of FOXO4 is significantly associated with low-grade lymph node metastases and stage III and IV tumors in colorectal cancer [27]. Another important function of FOXO4 is the role in cellular responses to oxidative stress. On one hand, FOXO4 regulates the cellular oxidative state by facilitating detoxification through the transcriptional activation of antioxidative enzymes, including superoxide dismutase and catalase. On the other hand, ROS modulate FOXO4 activity, either by activating the upstream regulatory pathways of FOXO4 or by sensing the cellular redox potential [28].

In the present study, we armed to the effects of 4-HNE on EVs formation, tumor cell growth, and migration, and we also found that FOXO4 activity positively correlated with 4-HNE levels. Under conditions of oxidative stress, whether 4-HNE interacts with FOXO4 to affect the biogenesis and development of EVs, thereby regulating the growth and metastasis of tumor cells, has not been reported. This is the focus of this study.

## Results

### The extracellular vesicles of lung cancer cells influence transfection efficiency and positively correlate with tumor growth

**Fig. 1:**
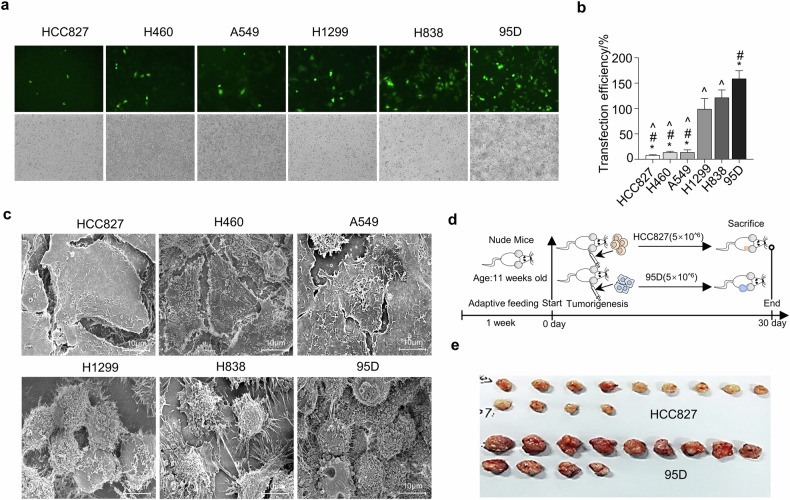
The transfection efficiency in lung cancer cell lines positively correlated with the number of extracellular vesicles and tumor cell growth. **a** Green fluorescent protein (GFP) expression in human lung cancer cell lines following transfection with the pEGFP-C1 vector via lipofection. The magnification was 200×. **b** Quantitative analysis of GFP expression levels across different groups, evaluated using one-way analysis of variance (*p < 0.05 versus H1299 group, ^#^*p* < 0.05 versus H838 group, ^*p* < 0.05 versus 95D group). **c** Scanning electron microscopy images revealing varied structural characteristics of cell membrane surface of human lung cancer cell lines. Scale bar = 10 μm. **d** HCC827 and 95D cells were subcutaneously implanted into the right flanks of nude mice, as detailed in the Methods section. **e** Tumors were excised and photographed 30 days post-injection.

As depicted in Fig. 1a, six lung cancer cell lines underwent transfection with the PEGFP-C1 vector by using liposomes. The number of green fluorescent cells per unit area was counted, and the result showed a notably higher green fluorescent protein (GFP) expression in 95D cells transfected with the pEGFP-C1 vector, in contrast to the lower expression observed in HCC827 cells (Fig. 1b). Concurrently, scanning electron microscopy (SEM) highlighted a morphological contrast; HCC827, H460, and A549 cells, characterized by a flat morphology, exhibited fewer EVs compared to the spherical H1299, H838, and 95D cells (Fig. 1c). These EVs are crucial for direct cellular connections, facilitating gap junctional intercellular communication and metabolic coupling among adjacent cells [29]. To further observations, HCC827 and 95D stable cell lines were subcutaneously inoculated into nude mice (Fig. 1d). Post 30 days, the development of subcutaneous tumors was assessed. Our findings indicate that the 95D cell line significantly accelerated tumor growth (Fig. 1e).

### Co-culturing of cells enhances information exchange and accelerates cell growth and migration

**Fig. 2:**
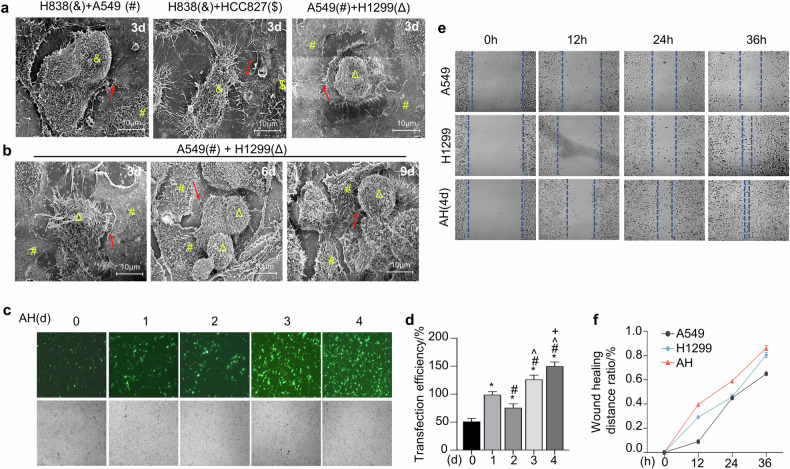
Accelerated effects of intercellular communication on migration and proliferation. **a** Scanning electron microscope (SEM) images showcasing structural interactions between co-cultured lung cancer cells over 3 days. H838 (&) cells mixed with A549 (#) cells, H838 (&) cells mixed with HCC827 ($) cells and A549 (#) cells mixed with H1299 (Δ) cells demonstrate intercellular connections (red arrows). Scale bar = 10 μm. **b** Time-course SEM images of A549 (#) and H1299 (Δ) cells co-cultured for 3, 6, and 9 days, highlighting progressive structural intercellular connectivity and increased formation of EVs (red arrows). Scale bar = 10 μm. **c** After co-culturing A549 and H1299 cells (referred to as AH) for 1, 2, 3, and 4 days, the cells were transfected with the PEGFP expression vector using lipofection. As the co-culture period increased, the transfection efficiency of the co-cultured cells also increased. the magnification was 100×. **d** Quantitative analysis of transfection efficiency, using one-way ANOVA reveals significant differences over time (*p < 0.05 versus 0 day group, ^#^*p* < 0.05 versus 1 day group, ^*p* < 0.05 versus 2 day group, ^+^*p* < 0.05 versus 3 day group). **e** The cell scratch assay images depicting the migration of A549, H1299, and co-cultured A549 + H1299 (referred to as AH) cells at 0, 12, 24, and 36 h. **f** Statistical evaluation of cell migration rates, expressed as a function of distance migrated over time, compares the migratory capacity of A549, H1299, and AH cells using one-way ANOVA.

To investigate whether cell lines with different morphologies can exchange information through EVs, thereby affecting cellular growth. We analyzed the cell surface morphology of co-cultured cells using SEM. The results showed that the two lung cancer cell lines could contact each other through EVs (Fig. 2a). A549 is an epithelial cell that was isolated from the lung of male with carcinoma. H1299 cell line was established from a lymph node metastasis of the lung from a patient who had received prior radiation therapy. Lymph nodes are frequent sites for lung cancer metastasis. the co-culture of A549 and H1299 cells is intended to simulate the lung cancer microenvironment more realistically. Here, we found there was an increase in the production of EVs between the A549 and H1299 co-cultured cells as co-culture time progressed (Fig. 2b). Following ‘AH cells’ refer to the co-cultured combination of A549 and H1299 cells. Next, the AH cells were transfected with the PEGFP-C1 vector. Statistical analysis indicated that the longer the cells were co-cultured, the higher the transfection efficiency in AH cells (Fig. 2c, d). In the cell scratch assay, the co-cultured AH cells also demonstrated a faster migration rate (Fig. 2e, f).

### Accelerated tumor growth in nude mice inoculated with co-culture cells

**Fig. 3:**
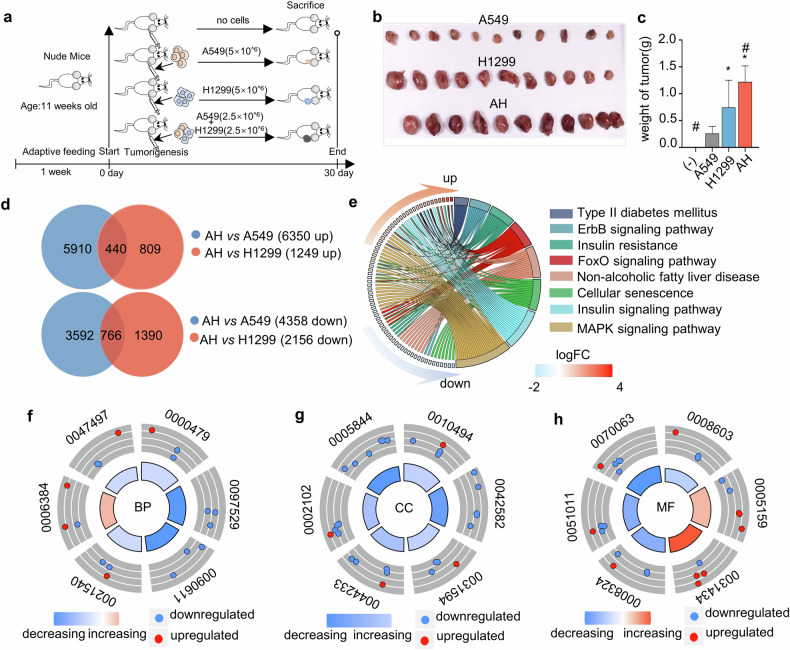
Mixed tumor cells accelerate tumor growth in tumorigenesis experiments. **a** A549 cells, H1299 cells, and co-cultured A549 + H1299 (referred to as AH) cells were subcutaneously implanted into the right flanks of nude mice, as outlined in the methods section. **b** Tumors were excised 30 days post-injection and photographed. **c** The weight of the tumors in each experimental group was statistically analyzed using one-way ANOVA (*p < 0.05 versus A549 group, ^#^*p* < 0.05 versus H1299 group). **d** Venn diagrams illustrate the number of upregulated and downregulated genes in tumor tissues comparing AH versus A549 and AH versus H1299 groups. Compared to A549 and H1299 groups, there are 440 upregulated and 766 downregulated genes in AH group. **e** KEGG pathway enrichment analysis of differentially expressed genes, with varying colors representing distinct pathways. *p* < 0.05 was considered statistically significant. **f**–**h** Gene ontology (GO) term distributions for biological process (BP), cellular component (CC), and molecular function (MF) categories are shown. Orange indicates upregulated genes, and blue indicates downregulated genes. *p* < 0.05 was considered statistically significant.

To investigate the biological effects of co-cultured cells on tumor growth in vivo, equal amounts of A549, H1299, and AH cells were suspended in saline and injected into nude mice (Fig. 3a). After 30 days, all three cell types had formed tumors in the mice. These tumors were then dissected, photographed, and weighed for analysis (Fig. 3b). There were significant statistical differences in tumor weight among the A549, H1299, and AH groups (Fig. 3c). Subsequently, tumor tissues from different groups were sent to a sequencing company for RNA microarray analysis. Based on the raw transcriptome data, we conducted venn analyses to identify genes that were exclusively upregulated or downregulated in the AH group compared to the A549 and H1299 groups. Specifically, there were 440 genes co-up-regulated (fold change >1.2, *p* < 0.05) in both AH vs A549 group (comprising 6350 up-regulated genes) and AH *vs* H1299 group (comprising 1249 up-regulated genes). Additionally, 766 genes were co-down-regulated (Fold Change >1.2, *p* < 0.05) in the comparisons of AH *vs* A549 group (4358 down-regulated genes) and AH *vs* H1299 group (2156 down-regulated genes), as illustrated in Fig. 3d.

To elucidate the potential biological functions of co-up-regulated and co-down-regulated genes (440 + 766), we employed the DAVID database for Kyoto Encyclopedia of Genes and Genomes (KEGG) and Gene Ontology (GO) functional enrichment analysis. KEGG pathway enrichment analysis showed that genes were mainly involved in 8 signaling pathways (Fig. 3e). In the AH tumor tissues, we identified 248 GO terms with *p* < 0.05. We visualized the top 6 ranked terms in biological processes (BP), cellular components (CC), and molecular functions (MF). The GO analysis revealed a significant enrichment of pathways related to the function of EVs. For instance, in BP, there were processes like ubiquitin-independent protein catabolic process via the multivesicular body sorting pathway, mitochondrion transport along microtubule (Fig. 3f). In CC, we observed ER-mitochondrion membrane contact site, Golgi-associated vesicle, multivesicular body membrane (Fig. 3g). In MF, cation transmembrane transporter activity, mitogen-activated protein kinase kinase binding (Fig. 3h). The abundant presence of pathways closely related to EVs in the GO functional enrichment analysis partially explains the relationship between tumor growth and EVs function in the AH group (Supplementary Table 3).

### Lipid peroxidation is closely related to the progression of NSCLC

**Fig. 4:**
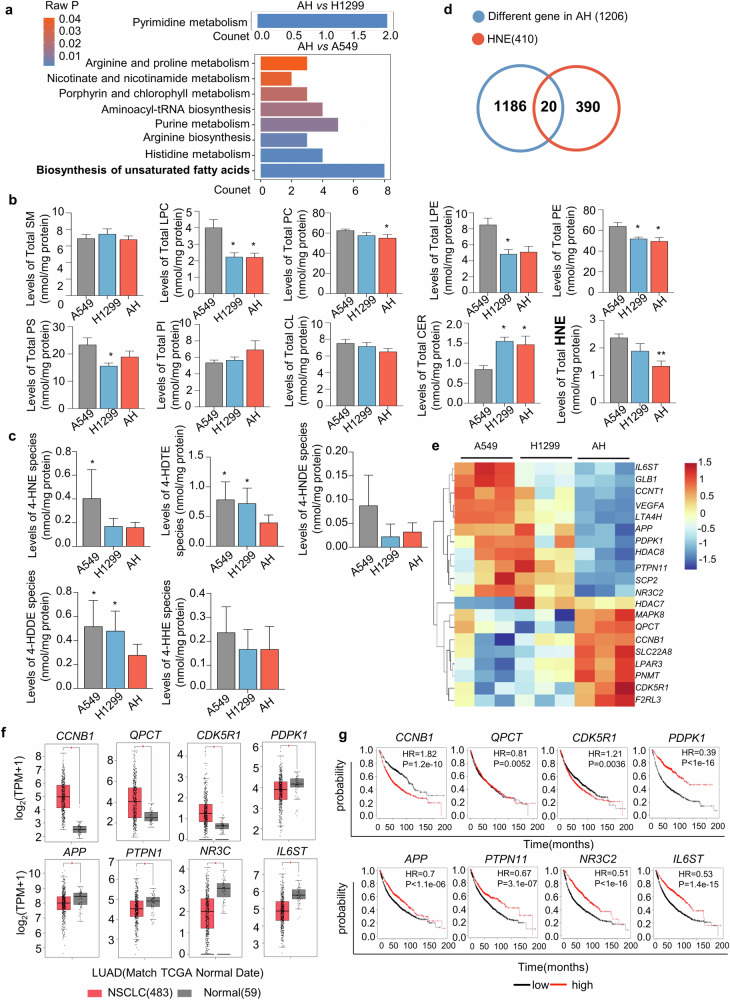
Reduced levels of lipid peroxidation product 4-HNE in mixed-cell formed tumors. **a** Histogram representation of metabolism pathway enrichment derived from differential metabolites between two groups. The top 8 pathways are listed on the right. Biosynthesis of unsaturated fatty acids changed most significantly. **b** Shotgun Lipidomics analysis of lipid extracts of tumor tissues from nude mice (*n* = 11) implanted by A549 cells (gray bar), H1299 cells (blue bar) and AH cells (red bar) groups was conducted by using multi-dimensional mass spectrometry-based shotgun lipidomics, with data analyzed by student’s unpaired t-tests, where *p < 0.05, **p < 0.01 versus A549 group. **c** Shotgun lipidomics analysis of HNE species in lipid extracts from tumor tissues in nude mice which were implanted with A549 cells, H1299 cells, and AH cells groups. With data analyzed by student’s unpaired t-tests, where *p < 0.05 versus AH group. **d** Venn diagram depicting the commonality between genes differentially expressed in AH cells and genes associated with HNE metabolism, identifying 20 genes shared between the two datasets. **e** Hierarchical clustering heatmap illustrating expression patterns of the 20 genes set among A549, H1299 and AH cells. **f** Validation of 20 genes against TCGA LUAD data via GEPIA; eight of the 20 overlapping genes showed significant correlation with NSCLC tumor, validating the reliability of the study findings. (*p < 0.01). **g** Overall survival analyses of genes (*CCNB1*, *QPCT*, *CDK5R1*, *PDPK1*, *APP*, *PTPN11*, *NR3C2*, and *IL6ST*) via Kmplot. *p* < 0.05 was considered statistically significant.

Metabolic transformation is a distinguishing feature of cancer, and the metabolism of cancer is governed by inherent cellular factors and the availability of metabolites within the TME [30]. In this study, a UPLC-Q/TOF-MS-based metabolomics approach was used to systematically evaluate metabolic transformation in tumor. Based on the statistical results from UPLC-Q/TOF-MS, the differential metabolites with a *p* < 0.05 (see Supplementary Tables 4–7) were subjected to pathway analysis through the MetaboAnalyst 5.0 website. The results revealed a significant enrichment in the biosynthesis of unsaturated fatty acids in the tumor tissues of the AH group (Fig. 4a, Supplymental Fig. 1a, b).

To identify the specific changes in lipid metabolites in the tumor, shotgun lipidomics was employed for the quantitative analysis of lipidomes in the tumor. Shotgun lipidomics analysis revealed that in the lipid extracts from tumor tissues of the AH group, the levels of sphingomyelin (SM) and phosphatidylcholine (PC) remained relatively stable; however, there was an increase in the content of phosphatidylinositol (PI) and ceramide (CER), while the levels of phosphatidylethanolamine (PE) and HNE decreased (Fig. 4b). Among these lipid metabolites, lipid peroxidation products of the HNE class have captured our attention. Compared to A549 tumors, the content of HNE was significantly reduced in H1299 tumors and further decreased in AH tumors. The results indicate that the level of HNE is inversely proportional to tumor growth rate (Fig. 4b). We conducted a precise analysis of the HNE compounds, and the results showed that the levels of 4-HNE, 4-hydroxy-dodecatrienal (4-HDTE), 4-hydroxy-2E,6Z-dodecadienal (4-HDDE) were the lowest in the tumors of the AH group (Fig. 4c). Lipid peroxidation initiates the formation of various reactive carbonyl compounds (RCCs), including HNE [31]. Briefly, 4-HNE, is generated by peroxidation of n-6 PUFAs, such as arachidonic, linoleic. 4-HDDE is exclusively produced from 12-lipxoygenase metabolites of arachidonic acid. 4-hydroxy-2E-hexenal (4-HHE) is the peroxidative product of n-3 PUFAs. Other HNE species, e.g., 4-hydroxynondienal (4-HNDE) and 4-HDTE can also be produced through peroxidation of PUFAs [32]. 4-HNE and 4-HDDE are not only reduced in H1299 group tumors, but are further decreased in AH group tumors, suggesting that the peroxidation of arachidonic acid is involved in regulating tumor growth.

To identify genes associated with HNE production in AH tumors, we conducted a search for the canonical SMILES structural formulae of HNE using PubChem. Both the STITCH website and the SwissTargetPrediction website were utilized to screen for datasets with target prediction capabilities. As a result, we obtained 410 target genes that are implicated in the regulation of HNE. We conducted a venn analysis between AH group’s differentially expressed genes (440 co-up-regulated and 766 co-down-regulated) and 410 HNE regulatory genes from these sites, identifying 20 shared genes (Fig. 4d). A heatmap analysis was conducted utilizing OmicStudio tools to illustrate the expression patterns of these 20 genes in A549, H1299, and AH group tumors (Fig. 4e).

Lung adenocarcinoma (LUAD) is the histological type of NSCLC. We analyzed the data of LUAD from the TCGA database on the GEPIA website for the aforementioned 20 genes. The results showed that among these genes, 8 genes exhibited statistically significant differential expression in NSCLC (Fig. 4f). Subsequently, we conducted survival analysis on these eight genes (Fig. 4g). The overall survival analysis by Kmplot indicated that high expression of *CCNB1* and *CDK5R1* in NSCLC cancer tissues is associated with poor prognosis in patients, while high expression of *PDPK1, PTPN11, NR3C2* and *IL6ST* in NSCLC cancer tissues is associated with improved prognosis in patients.

### The levels of 4-HNE and FOXO4 are reduced in AH group tumor and NSCLC samples

**Fig. 5:**
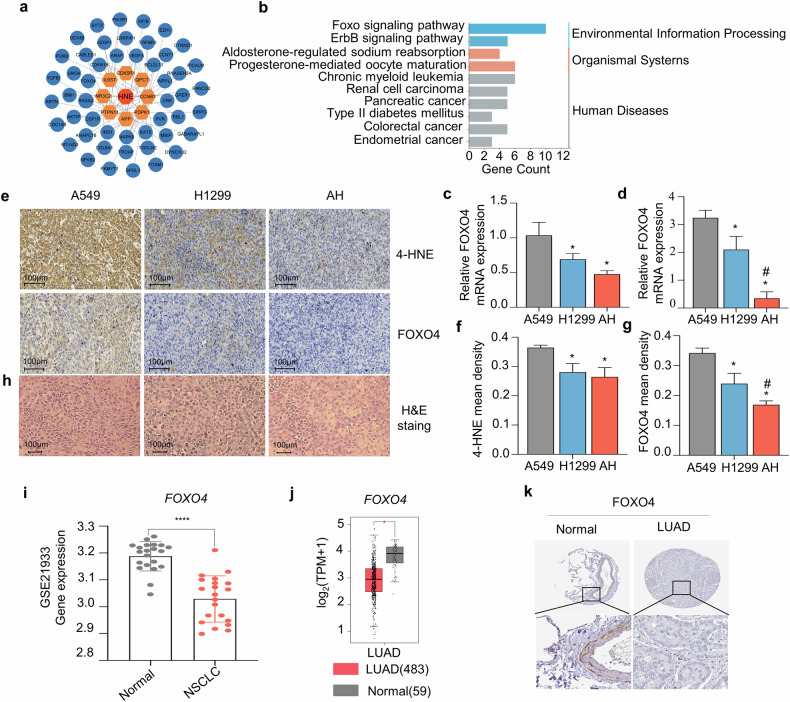
Decreased 4-HNE and FOXO4 levels in tumor tissues of the AH group and in NSCLC samples. **a** Protein-protein interaction (PPI) network illustrating connections between 58 proteins implicated in the regulation of HNE metabolism. **b** KEGG pathway analysis of 57 genes, conducted using the DAVID online database. **c** The relative mRNA expression levels of FOXO4 in tumor tissues inoculated with A549, H1299, and AH cells were quantified using RT-qPCR (*p < 0.05 versus A549 group). **d** Evaluation of the relative mRNA expression levels of FOXO4 in A549, H1299, and AH cells by RT-qPCR (*p < 0.05 versus A549 group, ^#^*p* < 0.05 versus H1299 group). **e** Immunohistochemical staining for 4-HNE and FOXO4 in tumor tissues, with representative images captured at 40× magnification. **f**, **g** Quantitative analysis of the stained positive area per field conducted using ImageJ software (NIH), with data presented as the mean of 9 fields ±SD (*n* = 3) and analyzed by one-way ANOVA (*p < 0.05 versus A549 group, ^#^*p* < 0.05 versus H1299 group). **h** Histopathological examination of mouse tumor tissues using H&E staining. Scale bar = 100 µm. **i** *FOXO4* gene expression validated in NSCLC versus normal tissue using the GSE21933 dataset, showing significantly reduced mRNA levels in NSCLC (****p < 0.0001). **j** Confirmation of *FOXO4* gene expression levels using TCGA LUAD data available in GEPIA. **k** Validation of FOXO4 protein expression in LUAD versus normal tissue using IHC data from The Human Protein Atlas.

To investigate the relationship between the 4-HNE and tumor growth, we analy association between the target genes of HNE and the 1206 genes (440 co-upregulated genes and 766 co-downregulated genes) by the STRING database. The interaction map of top 57 ranked proteins encoded by the genes were selected to construct a protein-protein interaction (PPI) network which involved in the regulation of HNE (Fig. 5a). Target genes that are highly interconnected with nodes in a module have been considered as functionally significant. Furthermore, the functional KEGG pathways of the 57 genes were explored using the DAVID online database, with the FOXO signaling pathway ranking first (Fig. 5b). We performed RT-PCR analysis on *FOXO4* genes and found that *FOXO4* expression was significantly downregulated in tumor tissues which were inoculated with AH cells (Fig. 5c). Meanwhile, when A549, H1299 and AH cells were cultured in vitro, *FOXO4* mRNA expression was also significantly downregulated in AH cells (Fig. 5d). Immunohistochemistry (IHC) results showed that compared to A549 group tumors, not only was the expression of FOXO4 and 4-HNE reduced in H1299 group tumors, but it was further decreased in AH group tumors (Fig. 5e–g). The hematoxylin and eosin (H&E) staining of tumor tissues showed that A549 group tumor cells had regular morphology with clear cell boundaries. H1299 group tumor cells exhibited irregular morphology, yet their cell boundaries were still relatively clear. In the AH group tumors, however, there were many cells with indistinct cell boundaries, which may be associated with the abundant formation of EVs among them (Fig. 5h).

To investigate the expression of *FOXO4* in NSCLC tumors, we analyzed the expression of *FOXO4* from the GEO database GSE21933, and the results showed a significant decrease in *FOXO4* mRNA in NSCLC tumors (Fig. 5i). We further analyzed the TCGA data and found the results is consistent (Fig. 5j). Furthermore, IHC staining data from the Human Protein Atlas database corroborate that FOXO4 protein expression in NSCLC tumors aligns with the transcriptional levels of FOXO4 in these tumors (Fig. 5k).

### 4-HNE and FOXO4 jointly reduce the formation of EVs and inhibit the growth and migration of co-culture lung cancer cells

**Fig. 6:**
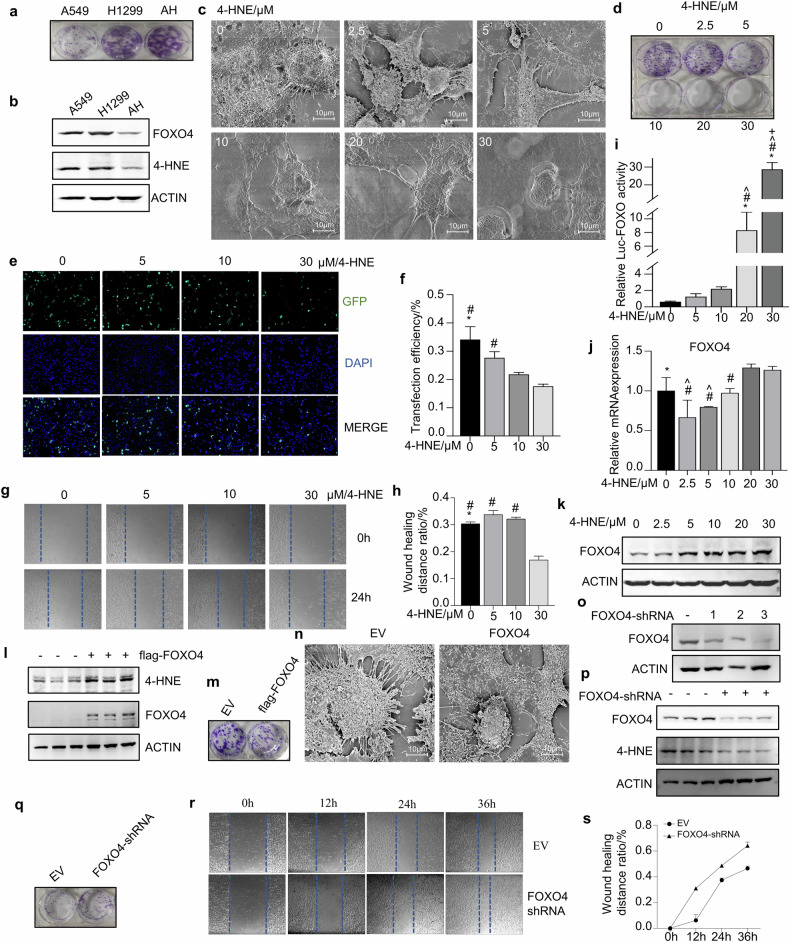
Impact of 4-HNE and FOXO4 on EV formation and the oncogenic attributes of co-cultured lung cancer cells. **a** Comparative analysis of colony formation in A549, H1299, and AH cell lines. **b** Quantification and relative expression levels of FOXO4 and 4-HNE proteins in A549, H1299, and AH cells by Western Blot Analysis. **c** The cell surface morphology of AH cells was observed by SEM after treatment with different concentrations of 4-HNE. **d** Colony formation post-4-HNE treatment: analysis of colony formation in AH cells across a range of 4-HNE concentrations (0, 2.5, 5, 10, 20, and 30 μM). **e** GFP expression in AH cells treatment with 4-HNE (0, 5, 10, and 30 μM) and subsequent transfected with pEGFP-C1 vector. Imaging at 100× magnification. **f** Quantitative analysis of the percentage of GFP-positive cells: statistical assessment across different treatment groups using one-way ANOVA. *p < 0.05 versus 10 μM group, ^#^*p* < 0.05 versus 30 μM group. **g**, **h** Cell scratch assay was used to determine the migration of AH cells treated with 4-HNE (0, 5, 10 and 30 μM) for 24 h. Statistical analysis of the migration rate in each group was performed using one-way analysis of variance (*p < 0.05 versus 5 μM group, ^#^*p* < 0.05 versus 30 μM group). **i** Luciferase reporter assay: transfection of FOXO4 expression plasmids and corresponding luciferase reporter vectors into 293 T cells, followed by 4-HNE treatment (0, 5, 10, 20, and 30 μM). Luciferase activity measured at 20 h post-treatment. Results are from three independent experiments; bar graphs represent mean ± SD. Significance marked as *p < 0.05 versus 0 μM group, ^#^*p* < 0.05 versus 5 μM group, ^*p* < 0.05 versus 10 μM grou*p*, ^+^*p* < 0.05 versus 20 μM group. **j** The mRNA expression of *FOXO4* in AH cells treated with 4-HNE (0, 2.5, 5, 10, 20, and 30 μM) were detected by RT-qPCR (*p < 0.05 versus 2.5 μM group, ^#^*p* < 0.05 versus 20 μM group, ^*p* < 0.05 versus 30 μM group). **k** Analysis of FOXO4 protein levels in AH cells treated with 4-HNE (0, 2.5, 5, 10, 20, and 30 μM) by Western Blot. **l** The expression of 4-HNE in AH cells which overexpressed FOXO4 was detected by western-blot analysis. **m** Comparative analysis of colony formation in AH cell lines. **n** Morphological examination of AH cells post-transfection with empty vector (EV) or FOXO4 vector by SEM. Scale bar = 10 μm. **o** A549 cells were transfected with three different FOXO4-targeted shRNAs, and western-blot analysis was used to evaluate the silencing efficiency of each shRNA. **p** Western-blot analysis assessed the expression levels of 4-HNE in A549 cells following FOXO4 silencing. **q** The impact of FOXO4 silencing on colony formation was examined in the A549 cell line. **r**, **s** The migration rate of A549 cells with silenced FOXO4 or not were assessed using a cell scratch assay. One-way ANOVA was employed to analyze the migration rates across different groups.

In this section, we explored the mutual regulation of FOXO4 and 4-HNE and examined their impacts on tumor cell proliferation, migration, and EVs formation. Colony formation assay results showed that compared to individual A549 and H1299 cells, the number of colonies significantly increased in cells co-cultured with both A549 and H1299 (Fig. 6a). The expression of FOXO4 and 4-HNE was significantly reduced in co-cultured AH cells (Fig. 6b), To assess the impact of 4-HNE on EVs formation, we exposed AH cells to varying concentrations of 4-HNE and subsequently conducted SEM observations. The results demonstrated that as the concentration of 4-HNE increased, there was a corresponding decrease in the number of EVs on the surface of AH cells (Fig. 6c). Concurrently, the results from the colony formation assay and lipofectamine transfection experiments demonstrated that with increasing concentrations of 4-HNE, there was a significant decrease in both colony formation capability (Fig. 6d) and transfection efficiency (Fig. 6e, f). Cell scratch assay demonstrated that with increasing concentrations of 4-HNE, there was a notable decrease in the migration (Fig. 6g, h). To further investigate the potential relationship between 4-HNE and FOXO4, a dual-luciferase reporter assay revealed that the transcriptional activity of FOXO4 enhanced in correlation with the rising concentrations of 4-HNE (Fig. 6i). Additionally, RT-qPCR results indicated that the mRNA levels of *FOXO4* increased with increasing concentrations of 4-HNE (Fig. 6j). A dose-dependent increase in the protein expression levels of FOXO4 was also observed when adding 4-HNE (Fig. 6k).

To further investigate the role of FOXO4, we overexpressed FOXO4 in AH cells. The results indicated an increase in 4-HNE protein levels (Fig. 6l) and a reduction in colony formation in the FOXO4 overexpression group (Fig. 6m). Additionally, to assess the impact of FOXO4 on EVs formation, we transfected AH cells with flag-FOXO4 expression plasmid and observed the EVs using scanning SEM method. SEM images revealed a decrease in the number of EVs in cells overexpressing FOXO4 (Fig. 6n). Given the significant reduction in FOXO4 expression in AH cells and its highest expression in A549 cells, we performed a knockdown of FOXO4 in A549 cells by using shRNA method. We initially screened for the most effective construction plasmid (Fig. 6o) and subsequently conducted Western blot analysis, colony formation assays, and scratch assays. The results demonstrated that FOXO4 knockdown led to a decrease in 4-HNE levels (Fig. 6p), an increase in the number of cell colonies (Fig. 6q), and enhanced cell migration rates (Fig. 6r, s).

## Discussion

In the past decade, EVs have emerged not only as important mediators of intercellular communication, but also as regulators of the microenvironment, playing a crucial role in maintaining homeostasis and influencing disease pathogenesis, including cancer metastasis [33]. There is a potential correlation between the invasiveness of NSCLC and the respective abilities of their EVs to induce cancerous phenotype [34]. Tumor-derived EVs promote cancer progression through changes in epithelial/endothelial barriers and immune regulation and includes the formation of pre-metastatic niches in distant sites. For instance, EVs from NSCLC cell lines (Calu6 and H358 cells) notably increased invasion and disrupted an epithelial barrier [35]. EVs from NSCLC also can expedite angiogenesis and tumor growth through a TGFβ1-dependent pathway [36].

We found the more EVs on the cell surface, the higher the efficiency of the cell in accepting liposome-transfected GFP vectors (Fig. 1a). When the mice inoculated with 95D which have more EVs exhibited significantly faster tumor growth (Fig. 1e). Cancer-derived EVs facilitate bi-directional communication between cancer cells and their surroundings that shape TME and contribute to cancer progression [37, 38]. EVs provide a critical link between primary tumors and the lymphatic inflammatory microenvironment, tumor-derived EVs have been demonstrated to disseminate rapidly through the lymphatic system into regional lymph nodes, particularly during infection [39]. Research indicates that EVs contribute to the establishment of a pre-metastatic niche--a conducive microenvironment within target organs that supports neoplastic implantation [40–42]. In this study, A549 and H1299 cells were co-cultured to simulate a more realistic TME. When two different cell lines are co-cultured, is there a promotion of their respective cell vitality through the exchange of EVs? We found the co-cultured cells exhibited faster growth rates and an increased production of EVs (Fig. 2a, b). The efficiency of lipid transfection increases with the duration of cell co-culture (Fig. 2c). In the tumor-forming experiment, we also observed that tumors formed from mixed cell inoculation exhibited the largest volume (Fig. 3b). Through metabolomic analysis, we found that the contents of lipid peroxidation products (4-HNE, 4-HDTE, 4-HDDE) (Fig. 4c) and *FOXO4* is significantly suppressed (Fig. 5c) in tumors of the AH group.

Oxidative stress is an intricate cellular state that governs the levels and content of released EVs. Cells release EVs to disseminate signaling molecules and respond to stress stimuli [9]. Since the early discovery of HNE’s ability to regulate the growth of malignant cells, HNE has been described as a biphasic growth factor, being stimulatory at low doses and inhibitory/cytotoxic at high doses [43]. Prooxidants would lead to the formation of lipid-peroxyl radicals (ROO•), lipid peroxides (ROOH), and reactive aldehydes like 4-HNE and malondialdehyde (MDA) [44]. When lipid peroxidation occurs, it alters the physical properties of the membrane, affecting phospholipid dynamics, membrane shedding, fluidity, and permeability [45]. 4-HDTE and 4-HDDE have structures like 4-HNE, all of them are the products of lipid peroxidation induced by oxidative stress. 4-HNE can form covalent adducts with proteins, leading to structural changes in proteins, so it has been described as a ‘second messenger’ in various cellular signaling pathways [45, 46]. In hepatocellular carcinoma, the expression of 4-HNE is stronger in adjacent tissues than in the tumor. Similarly, stronger expression of 4-HNE in adjacent tissues has also been observed in metastatic lung cancer, supporting the hypothesis that non-malignant cells near the cancer produce 4-HNE to protect themselves from the invading cancer cell [47, 48]. Recently, 4-HNE accumulation has been linked to ferroptosis, characterized by increased intracellular redox-active iron and impaired lipid peroxide repair capacity [49, 50]. In this study, the significant reduction of lipid peroxidation products such as 4-HNE, 4-HDTE and 4-HDDE (Fig. 4c) in the AH group tumors suggests that inhibiting lipid peroxidation contributes to the formation of EVs and the growth of tumors.

By analyzing differentially expressed genes which are associated with the generation of HNEs in AH group tumor, FOXO4 as a target has caught our attention. IHC results reveal that both FOXO4 and 4-HNE exhibit the lowest expression in AH group (Fig. 5e). FOXOs are generally considered tumor suppressors due to their well-known functions in cell cycle arrest, apoptosis, DNA damage repair, and scavenging of ROS [22]. Several studies have shown an association between reduced FOXO4 expression and heightened cancer development. Low expression of the FOXO4 contribute to the epithelial-mesenchymal transition (EMT) in NSCLC and miR-150 promotes cellular metastasis in NSCLC by targeting FOXO4 [23, 51]. FOXO4 was also downregulated in colorectal cancer tissues compared with normal tissues. Overexpressed FOXO4 suppressed EMT and the migration of colorectal cancer cell lines [24]. Exosomal miR-128-3p overexpression could downregulate the expression of FOXO4 in colorectal cancer cells, which led to EMT [52]. We analyzed data from the GEO database and also found that *FOXO4* is significantly downregulated in NSCLC cancer tissues (Fig. 5i). Low expression of *FOXO4* is associated with worse prognosis in NSCLC cancer patients.

As is well known, the rapid growth of tumors often leads to the formation of a hypoxic microenvironment, subsequently triggering oxidative stress reactions. EVs released under oxidative stress contain antioxidant molecules that regulate oxidative stress reactions in target cells, thereby protecting cells from further damage [53, 54]. The abundant generation of EVs may be a crucial mechanism ensuring rapid tumor growth. In this study, when 4-HNE is added to cells or when FOXO4 is overexpressed, the number of EVs on the cell surface significantly decreased. Cellular experiments showed that 4-HNE addition to cultured cells increases FOXO4 expression and transcriptional activity. In line with this, FOXO4 overexpression elevated 4-HNE protein levels and reducted colony formation. Furthermore, FOXO4 knockdown decreased 4-HNE levels, increased colony formation and enhanced cell migration rates (Fig. 6). FOXO4 has been found to cause cell cycle arrest in number of studies [55, 56]. Therefore, maintaining a low content of 4-HNE and FOXO4 in tumor tissues may be an important way to sustain tumor rapid growth.

In summary, we report when two different NSCLC cells are co-cultured, they exchange information through EVs, which promotes the deterioration of the cells. During this process, the levels of both 4-HNE and FOXO4 are downregulated. The activity of FOXO4 is positively correlated with the levels of 4-HNE. Both 4-HNE and FOXO4 can inhibit the formation of EVs. Therefore, modulating the production of 4-HNE and the activity of FOXO4 may be an effective strategy for the treatment of NSCLC.

## Materials and methods

### Chemical and biochemical materials

DMEM (C11995500BT, Gibco), FBS (086-150, Wisent corporation). Lipofectamine 2000 (11668019, ThermoFisherScientific), flag-FOXO4 (17549, Addgene), Foxo NLuc luciferase reporter (178318, Addgene), pRL Renilla Luciferase Control Reporter Vector (E2231, Promega), Dual-Luciferase® reporter assay system (E1910, Promega). PrimeScript™ RT Master Mix (Perfect Real Time) (RR036A, TaKaRa), TB Green^®^ PremixExTaq™ (Tli RNaseHPlus) (RR420A, TaKaRa). 4-HNE (HY-113466, MedchemExpress), Rabbit Anti-4 Hydroxynonenal antibody (bs-6313R, Bioss Antibodies). FOXO4 Polvclonal antibody (21535-1-AP, Proteintech), β-actin Antibody (C4) (sc-47778, Santa cruz), IRDye 800CW Goat anti-Rabbit IgG (925-32211, LICOR), IRDye 680RD Goat anti-Mouse IgG (H + L) (926–68070, LICOR).

### Cell culture

HCC827, NCI-H460, A549, NCI-H1299, NCI-H838 (all human non-small cell lung carcinoma cells) were obtained from ATCC, and 95D (highly metastatic lung cancer cells) were obtained from our laboratory backup. These six cell lines were maintained in DMEM supplemented with 10% FBS, incubated at 37 °C in a 5% CO_2_ environment. For co-culturing, equal numbers of cells were combined and cultured. AH cells used in experiments were co-cultured for 2 weeks. All cells were regularly tested for mycoplasma cytoplasmic contamination.

### Cell transfection and fluorescence observation

Different cells were seeded in 6-well plates at 2.5 × 10^5^ cells per well. Transfection was performed with PEGFP-C1 vector using Lipofectamine 2000, following the manufacturer’s instructions. 36 h post-transfection, DAPI was used for nuclear staining. Fluorescence microscopy imaging was conducted using an Axio Observer A1 inverted microscope (AX10, Zeiss). The efficiency of liposome transfection was determined by calculating the proportion of cells emitting green fluorescence in the same number of cells with using ImageJ. Results are expressed as mean ± SD from three independent experiments [39].

The shRNA plasmid targeting FOXO4 was constructed using the PGMLV-hU6-MCS-CMV-ZsGreen1-PGK-Puro-WPRE vector (2494-PGMLV- SC5) provided by Jiman Biotechnology Company. Based on the FOXO4 gene sequence, three appropriate target sequences for shRNA were selected (Table 1). The corresponding shRNA oligonucleotides (Table 2) were synthesized and then three shRNA plasmids were constructed and packaged into lentiviruses using the GM easyTM lentivirus packaging kit (GMeasy-10, Genomeditech), following the manufacturer’s instructions. Cells infected with these lentiviruses were subsequently detected using Western blot assays to identify those with the most effective knockdown for use in further experiments. Specific target sequences and the synthetic shRNA oligo sequences are detailed in Tables 1 and 2.

**Table 1:** The shRNA sequence for FOXO4 target.

NO.	TargetSeq
NC	TTCTCCGAACGTGTCACGT
shRNA1250	ACCGTGAAGAAGCCGATATGT
shRNA1914	TCAGGATCTAGATCTTGATAT
shRNA2318	CACTTAGGCTTTGTAGCAAGA

**Table 2:** The shRNA oligo sequence for constructing plasmid.

Oligo name	Oligomeric single-stranded DNA sequence 5 ‘to 3'
Primer-NC-T	GATCTGTTCTCCGAACGTGTCACGTTTCAAGAGAACGTGACACGTTCGGAGAATTTTTTC
Primer-NC-B	AATTGAAAAAATTCTCCGAACGTGTCACGTTCTCTTGAAACGTGACACGTTCGGAGAACA
Primer-T1	GATCCACCGTGAAGAAGCCGATATGTCTCGAGACATATCGGCTTCTTCACGGTTTTTTT
Primer-B1	AATTAAAAAAACCGTGAAGAAGCCGATATGTCTCGAGACATATCGGCTTCTTCACGGTG
Primer-T2	GATCCGTCAGGATCTAGATCTTGATATCTCGAGATATCAAGATCTAGATCCTGATTTTTT
Primer-B2	AATTAAAAAATCAGGATCTAGATCTTGATATCTCGAGATATCAAGATCTAGATCCTGACG
Primer-T3	GATCCGCACTTAGGCTTTGTAGCAAGACTCGAGTCTTGCTACAAAGCCTAAGTGTTTTTT
Primer-B3	AATTAAAAAACACTTAGGCTTTGTAGCAAGACTCGAGTCTTGCTACAAAGCCTAAGTGCG

### Scanning electron microscope (SEM)

Cell samples were washed twice with PBS and fixed in 2.5% glutaraldehyde at 4 °C overnight to preserve morphology. They were then rinsed thrice with deionized water for 10 min each at room temperature. Gradual dehydration was performed to replace cellular water content with ethanol, which has a lower surface tension than water, preventing deformation in the SEM vacuum chamber. This dehydration process involved increasing ethanol concentrations: 50%, 70%, 90%, and 100% (v/v) at room temperature. Then samples were processed in a 50% tert-butyl alcohol and 50% ethanol mixture for 10 min, followed by 100% tert-butyl alcohol, and then cooled to 4 °C. The final step involved drying the samples in a vacuum before SEM observation using a Hitachi SU8010.

### Cell scratch assay

Cells were cultured in 6-well plates to approximately 90% confluence. A 20 μL sterile pipette tip was used to create a scratch in the cell monolayers. After scratching, the cells were washed with PBS and then incubated in DMEM containing 1% FBS. At 12, 24, and 36 h post-scratch, images of the scratch area were captured using an inverted fluorescence microscope (Zeiss AX10). Wound area calculated by manually tracing the cell-free area in captured images using the ImageJ. Under normal conditions, the wound area will decrease over time. The migration rate can be expressed as the percentage of area reduction or wound closure. The closure percentage will increase as cells migrate over time [57]:

$${\rm{Wound\; Closure}} \% :\{({{\rm{A}}}_{{\rm{t}}=0{\rm{h}}}-{{\rm{A}}}_{{\rm{t}}=\triangle {\rm{h}}})/{{\rm{A}}}_{{\rm{t}}=0{\rm{h}}}\}\times 100 \%$$

A_t=0h_ is the area of the wound measured immediately after scrating(t = 0 h)A_t=∆h_ is the area of the wound measured h hours after the scratch is performed

### Tumor formation experiment in nude mice

11-week-old C57BL/6J female nude mice were acquired from the Zhejiang Chinese Medical University Animal Center. All procedures complied with the ethical guidelines of the Zhejiang Chinese Medical University’s Committee for Experimental Animal Use. All nude mouse experiments were used by single blind method. The number of mice used in mouse tumorigenesis experiments depends on the experimental design, study purpose, and statistical power analysis. In general, in order to ensure the statistical significance and repeatability of experimental results, at least 5 to 10 mice are required in each experimental group. Refer to ARRIVE Guide [58], the number of mice selected by us all meet the statistical requirements of mouse tumor formation test.

The first group consists of 26 nude mice randomly divided into 2 groups by paired comparison method, with 13 mice in each group, inoculated with HCC827 and 95D cells, respectively. The second group consists of 44 nude mice randomly divided into 4 groups by randomized block method, with 11 mice in each group, inoculated with A549 cells, H1299 cells, and AH cells or saline solution. Each mouse in the experimental groups received a subcutaneous injection of 0.2 mL saline solution containing 5 × 10^6^ cells in the flank. Control mice received a 0.2 mL saline solution injection without cells. The mice were housed at 22–25 °C and 60–70% humidity, with ad libitum access to water and food.

After 30 days, mice from each group were euthanized. Tumors were rinsed with PBS until blood-free and stored at −80 °C. The tissues underwent UPLC-Q/TOF-MS analysis, lipidomics analyses, mRNA microarray analysis and histopathological evaluation.

### UPLC-Q/TOF-MS analysis

6 transplanted tumor tissue were randomly selected in A549, H1299, and AH cells groups for the untargeted metabolomics analysis. 10 mg of transplanted tumor was added to 20 μL ice ultrapure water and 180 μL mixture of 20% methanol solution / 80% acetonitrile, vortexed and shaken, and left to stand in room temperature for 10 min. Then centrifuged (14000 r/min for 20 min at 4 °C), and the supernatant was collected to desiccation and diluted with mixture of ultrapure water and methanol solution/acetonitrile (2/8) in a ratio of 1:1. After that, the supernatant was collected and centrifuged for 20 min at 14,000 r/min and 4 °C to obtain supernatant of a tissue homogenate for untargeted metabolomic analysis using UPLC-Q/TOF-MS.All samples were used as the quality control (QC) samples. The specifc chromatographic and mass spectrometric conditions and data processing and analysis were performed according to the previously published article [59]. Here, we use the Gradient which consisted of 95% eluent B, 0–1 min; 95–1% eluent B, 1–20 min; 1% eluent B, 20–23 min. Univariate statistical analysis with *p* < 0.05 was used to screen metabolites with significant differences in A549 group, H1299 group, and AH group, and The MetDNA website (http://metdna.zhulab.cn), OSI/SMMS software (http://www.5omics.com) and the online HMDB (http://www.hmdb.ca/), METLIN (http://metlin.scripps.edu/), Lipid Maps (www.lipidmaps.org) databases were used for substance identification (ppm < 5). Metabolite pathway analysis was performed using MetaboAnalyst 5.0 (https://www.metaboanalyst.ca).

### Shotgun lipidomics analysis of tumor tissues

For shotgun lipidomic analysis, tumor tissues were examined using a triple-quadrupole mass spectrometer (Thermo TSQ Quantiva) equipped with an automated nanospray ion source (TriVersa NanoMate, Advion Bioscience Ltd.). The testing of the samples was carried out by our platform’s laboratory technicians who specialize in operating this instrument. Briefly, a revised protocol was used for lipid extraction from tumor tissues [60, 61]. Lipid extracts were resuspended in 1000 μL of chloroform/methanol (1:1)/mg protein and stored at −20 °C for mass spectrometry (MS) analysis [62]. Derivatization of HNE was performed followed previously established methods [63]. Lipid species were identified through multi-dimensional MS and mass spectra were acquired using custom sequence subroutines in Xcalibur software and [64]. Data were presented as mean ± standard error of the mean (SEM). Statistical differences between groups were assessed using Student’s unpaired t-tests. Analysis of HNE was completed within 7 days, and other analyses within 2 weeks.

### mRNA microarray analysis

Total RNA from A549, H1299, and AH group tumors was extracted using Trizol. RNA quality and quantity were determined using a NanoDrop ND-1000 spectrophotometer, and integrity was verified via standard denaturing agarose gel electrophoresis. The samples were processed at Kangchen Bio-tech company by using the Agilent Human 4 × 44K chip array. Differential gene expression analysis was performed using Fold Change filtering. The array data is accessible under GEO accession number GSE124112.

Upregulated and downregulated genes in AH vs A549 and AH vs H1299 were collected, using a filter of |FC | > 1.2 and *p* < 0.05. Venn analysis identified overlap genes that were either upregulated or downregulated. To explore the function of intersecting genes, GO enrichment analysis and KEGG pathway analysis were performed using the DAVID online database. The gene lists were uploaded for analysis, yielding results in biological process (BP), cellular component (CC), molecular function (MF), and KEGG pathways.

### Bioinformatics analysis

Prediction of HNE-regulated target genes: HNE is inputted into the PubChem website to obtain the standardized SMILES structure formula. STITCH 5.0 predictions classify manually curated information and experimentally validated protein-chemical interactions, and in this study, we considered chemical interactions with a STITCH confidence score of ≥0.4 (ref. [65]). Swiss Target Prediction is a web server for identifying biological targets of bioactive small molecules, combining 2D and 3D similarity metrics to predict the top 15 targets [66]. We entered HNE’s SMILES structure formula into the STITCH website and SwissTargetPrediction website to search for protein information related to HNE regulation.

Heatmap and PPI Network Construction: Gene expression in tumor tissues was analyzed using OmicStudio’s heatmap tool. The STRING database is an online resource whose main function is to construct functional protein association networks [67]. Differentially expressed genes from AH tumors were entered into the STRING database to identify those most closely associated with direct regulation of the HNE gene. Interactions with scores >0.40 were considered significant, leading to the identification of 58 core targets. The PPI network for these 58 target genes was constructed using Cytoscape software, where nodes represent genes and edges represent protein interactions.

Gene expression and survival correlation analysis in tumors: GEPIA2 is a Web server for analyzing the RNA sequencing expression data of 483 NSCLC and 59 normal samples from the TCGA and the TCGA projects, using a standard processing pipeline. Eight target genes (*p* < 0.05) were chosen as the candidates for further analysis. Survival analysis was conducted using the online database Kmplot, focusing on patient survival percentages over time. Patients were classified based on k-means clustering, and the analysis was executed using the R package “survival”. The log-rank test assessed statistical differences between survival curves, with *p* < 0.05 indicating statistical significance. The GSE21933 dataset, which includes gene expression data from tumor of 21 NSCLC patients and normal samples, was retrieved from the GEO database. GraphPad Prism 8 was used to analyze and identify differentially expressed genes through one-way ANOVA. The translational-level validation of genes was carried out using The Human Protein Atlas database. Network Pharmacology database informationare detailed in Table 3.

**Table 3:** Network Pharmacology database information table.

Database	URL
PubChem	https://pubchem.ncbi.nlm.nih.gov/
Stitch	http://stitch.embl.de
Swiss Target Prediction	http://swisstargetprediction.ch
OmicStudio’s heatmap	https://www.omicstudio.cn/tool
String	https://cn.string-db.org/
GEPIA2	http://gepia.cancer-pku.cn/
Kmplot	http://kmplot.com/analysis/
GEO	http://www.ncbi.nlm.nih.gov/geo
DAVID	https://david.ncifcrf.gov/summary.jsp
Human Protein Atlas database	https://www.proteinatlas.org/

### Reverse transcription-quantitative PCR (RT-qPCR)

Cells were cultured in 6-well plates and total RNA was extracted using TRIzol, following the manufacturer’s protocol. After RNA quantification, reverse transcription was performed using PrimeScript™ RT Master Mix to synthesize cDNA. Target gene quantification was conducted using TB Green® Premix Ex Taq™ (Tli RNaseH Plus), with GAPDH serving as the internal control. The relative mRNA expression levels were calculated using the 2^-ΔΔCT^ method. Primers for gene amplification included:

*FOXO*4-F, 5-GCCAAGACAGAATGCCTCAGGATC-3;

*FOXO4-R*, 5-GTCCAGTCCCTCGCCCTCATC-3;

*GAPDH-F*, 5-TGACATCAAGAAGGTGGTGAAGCAG-3;

*GAPDH-R*, 5-GTGTCGCTGTTGAAGTCAGAGGAG-3.

### Histopathology and immunohistochemistry

Tumor tissues were fixed in 4% paraformaldehyde and embedded in paraffin following standard procedures. Serial sections were prepared and subjected to H&E staining, as well as IHC. For IHC, primary antibodies (Rabbit Anti-4-HNE and FOXO4 Polyclonal antibody) were applied. The staining was developed using DAB substrate solution after incubation with secondary antibody and biotin-streptavidin HRP conjugate. Imaging of all samples was performed using a KFBIO digital white light scanner (KF-PRO-120).

### Dual-luciferase reporter assay

293 T cells were seeded in 48-well plates at 0.5 × 10^5^ cells per well and cotransfected with 0.3 μg of firefly luciferase reporter (Luc-FOXO), 0.3 μg of flag-FOXO4, and 0.03 μg of Renilla luciferase reporter using Lipofectamine 2000. After 24 h of transfection, cells were treated with 4-HNE (5, 10, 20, and 30 μM) or left untreated. Twenty hours post-treatment, cell lysates were prepared for luciferase reporter activity analysis using the Dual-Luciferase Reporter Assay System kit. Results are expressed as mean ± SD from three independent experiments.

### Cell colony formation assay

Cells were seeded in 6-well plates at a density of 400 cells per well, in triplicate. The cells were cultured at 37 °C in a 5% CO_2_ environment for 14 days, with medium changes every 3 days. Post-incubation, cells were fixed with 4% paraformaldehyde, stained with 0.5% crystal violet, and cell colony morphologies were assessed.

### Western-blotting assay

Cells were lysed using RIPA buffer to extract proteins. Protein concentrations were determined using the BCA Protein Assay Kit (P0010, Beyotime). Equal amounts of protein were combined with 2x SDS loading buffer, heated at 100 °C for 10 min, separated by 10% SDS-PAGE, and transferred onto PVDF membranes. These membranes were incubated overnight at 4 °C with primary antibodies (rabbit anti-4-HNE, FOXO4 polyclonal, or β-actin antibody C4), followed by 2 h with corresponding secondary antibodies at room temperature. Blots were visualized using an Odyssey scanner (LI-COR), quantification of Western blot signal was done by densitometry using ImageJ.

### Statistical analysis of data

The data shown represent at least three independent experiments and are expressed as mean ± SEM. Statistical analyses were conducted using GraphPad Prism 8. *p* values were calculated using one-way ANOVA and Student’s unpaired *t* tests, with a *p* value of less than 0.05 considered statistically significant.

### Supplementary information

Supplemental Information

Uncropped western blots

## Data Availability

Original images of immunoblotting are provided as supplementary material. The accession number for all raw sequencing data reported in this paper is available at GEO: GSE124112. All other datasets generated in this study are presented and analysed within this manuscript and are available from the corresponding authors upon request.
