# Supplementary material for: Downregulation of 4-HNE and FOXO4 collaboratively promotes NSCLC cell migration and tumor growth
Source: Cell Death Dis. 2024 Jul 31;15(7):546. doi: 10.1038/s41419-024-06948-4 (PMC11291900; doi:10.1038/s41419-024-06948-4)
Supplement: Supplementary file 1 — Supplemental Information [file 41419_2024_6948_MOESM1_ESM.doc]

**Supplemental Information**

**Downregulation of 4-HNE and FOXO4 Collaboratively** **Promotes NSCLC Cell Migration and Tumor Growth**

**Tianfei Zhong** ^1, 2, #^, **Ying Li** ^3, #^, **Meng Jin** ^3, #^, **Jingqun Liu** ^3^, **Zhenyu Wu** ^1, 4^, **Feiye Zhu** ^5^, **Lisha Zhao** ^6^, **Yongsheng Fan** ^4, 7^, **Li Xu** ^1, 4^ *, **Jinjun Ji** ^1, 4^ *

1. College of Basic Medical, Zhejiang Chinese Medical University, 548 Binwen Road, Hangzhou, 310053, China
2. Logistic Affairs Department, Zhejiang Chinese Medical University, 548 Binwen Road, Hangzhou, 310053, China
3. The First School of Clinical Medicine, Zhejiang Chinese Medical University, 548 Binwen Road, Hangzhou, 310053, China
4. Key Laborat Laboratory of Chinese Medicine Rtheumatology of Zhejiang Province, 548 Binwen Road, Hangzhou, 310053, China
5. Academy of Chinese Medical Sciences, Zhejiang Chinese Medical University, 548 Binwen Road, Hangzhou, 310053, China
6. Department of Medicine, Zhejiang Academy of Traditional Chinese Medicine, Hangzhou, 310007, China
7. Department of Rheumatology, The Second Affiliated Hospital of Zhejiang Chinese Medical University, Hangzhou, 310053, China

**^#^These authors contributed equall**y: Tianfei Zhong, Ying Li, Meng Jin

***Correspondence**: Jinjun Ji, Tel: +86 18957130151, Email: [jijinjun@zcmu.edu.cn;](mailto:jijinjun@zcmu.edu.cn;) [Li](mailto:gregorylazar@gmail.com;Li) Xu, Tel: +86 13003691133, Email: [xulihhb@163.com](mailto:xulihhb@163.com)

This information includes: Supplementary Figure 1 a-b

Supplementary Tables 1-8


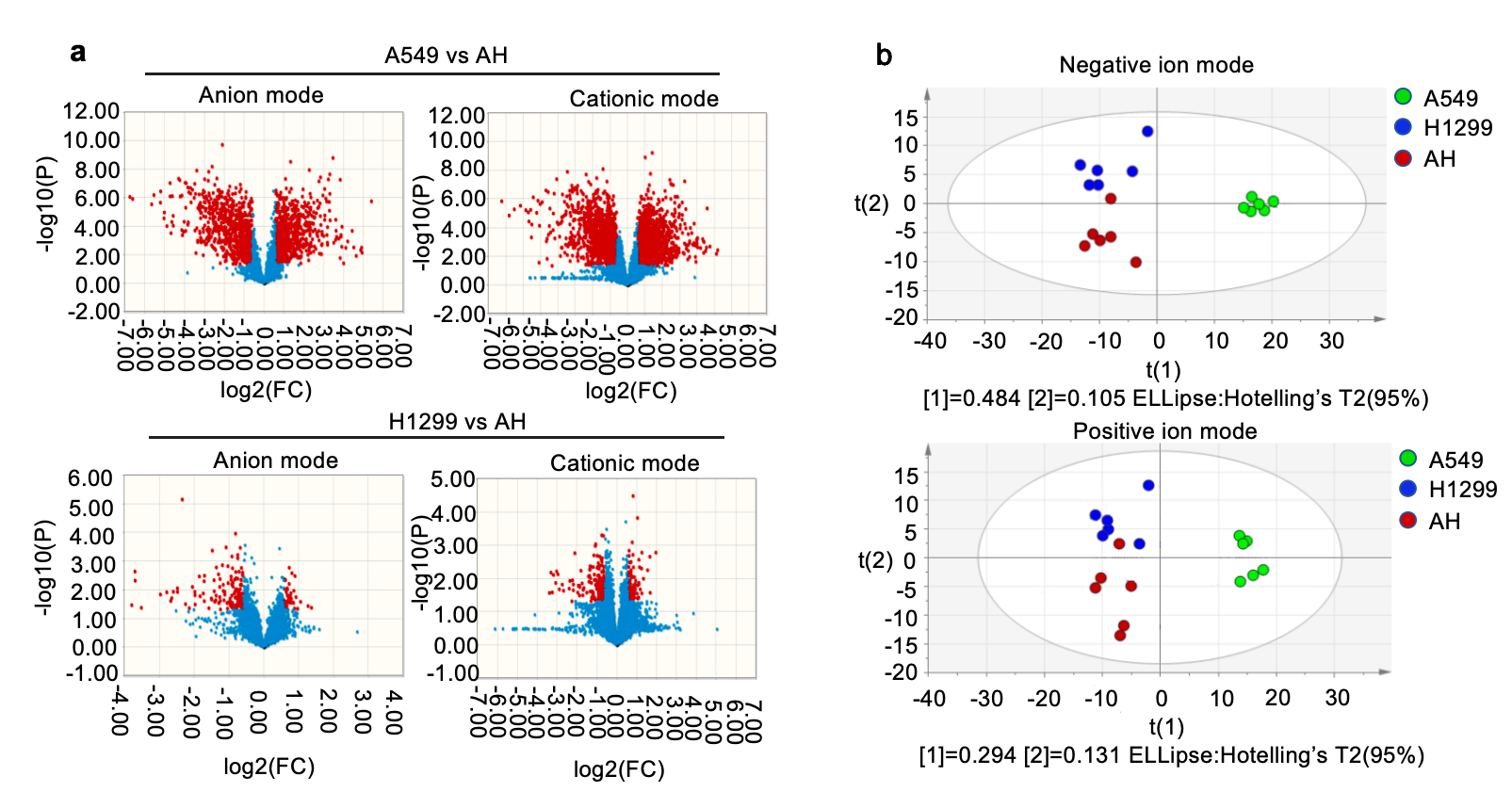


Supplementary Fig.1 **a** The volcano plot of the positive and negative ion mode data in different samples. The abscissa represents the fold change of metabolites in different groups (log2 Fold Change), the ordinate represents the significant level of difference (-log10 *p*-value), and each point in the volcano map represents a metabolite. **b** The SIMCA positive-derived and negative-derived PLS-DA among the A549, H1299, and AH groups.

**Supplementary Table 1.** List of abbreviations for lipid metabolites

| **Abbreviation** | **Full Name** |
| --- | --- |
| LPE | Lysophosphatidyl ethanolamine |
| LPC | Lysophosphatidylcholine |
| SM | Sphingomyelin |
| PE | Phosphatidylethanolamine |
| PC | Phosphatidylcholine |
| PS | Phosphatidylserine |
| HNE | 4-hydroxyalkenals |
| PI | Phosphatidylinositol |
| CL | Cardiolipin |
| CER | Ceramide |

**Supplementary Table 2.** List of abbreviations for lipid metabolites

| **Abbreviation** | **Full Name** |
| --- | --- |
| 4-HNE | 4-hydroxy-2E-nonenal |
| 4-HDTE | 4-hydroxy-dodecatrienal |
| 4-HNDE | 4-hydroxy-nondienal |
| 4-HDDE | 4-hydroxy-2E,6Z-dodecadienal |
| 4-HHE | 4-hydroxy-2E-hexenal |

**Supplementary Table 3.** GO functional enrichment analysis

| **ID** | **Term** | **Genes** | **Category** | **adj_pval** |
| --- | --- | --- | --- | --- |
| GO:0000479 | endonucleolytic cleavage of tricistronic rRNA transcript (SSU-rRNA, 5.8S rRNA, LSU-rRNA) | RCL1, BMS1, TSR1 | BP | 0.006462 |
| GO:0097529 | myeloid leukocyte migration | NF1, PIK3R1, PAFAH1B1 | BP | 0.012520 |
| GO:0090611 | ubiquitin-independent protein catabolic process via the multivesicular body sorting pathway | PDCD6IP, CHMP4C, CHMP4B | BP | 0.020217 |
| GO:0021540 | corpus callosum morphogenesis | SZT2, NIN, PAFAH1B1 | BP | 0.020217 |
| GO:0006384 | transcription initiation from RNA polymerase III promoter | SNAPC5,GTF3C4, BRF1 | BP | 0.039870 |
| GO:0047497 | mitochondrion transport along microtubule | UXT, MAP1B, LRPPRC | BP | 0.039870 |
| GO:0042582 | azurophil granule | SNAP23, ANXA1, AZU1, STX3 | CC | 0.009215 |
| GO:0044233 | ER-mitochondrion membrane contact site | AHCYL1, MBOAT7, VPS13A, CLCC1, TOMM20 | CC | 0.014397 |
| GO:0005798 | Golgi-associated vesicle | APP, STK16, MAP6, NUCB1 | CC | 0.04766 |
| GO:0032585 | multivesicular body membrane | LAPTM4B, CHMP4C, CHMP4B, ATP13A2, TMEM9 | CC | 0.038232 |
| GO:0002102 | podosome | SVIL, HNRNPK, DBNL, CTTN, LPXN | CC | 0.047629 |
| GO:0005844 | polysome | UPF2, FUBP3, DHX9, RPS6, MSI2, DIS3L2 | CC | 0.026544 |
| GO:0008603 | cAMP-dependent protein kinase regulator activity | PRKAR1B, PRKAR1A, PRKAG1 | MF | 0.044312 |
| GO:0005159 | insulin-like growth factor receptor binding | SOCS2, SOCS1, IRS1, PIK3R1, CRK | MF | 0.006963 |
| GO:0031434 | mitogen-activated protein kinase kinase binding | TAOK2, TRIB3, TRIB2, MAP3K11 | MF | 0.024430 |
| GO:0008324 | cation transmembrane transporter activity | SLC30A7, SLC30A5, SLC30A1, PKD2L1 | MF | 0.029961 |
| GO:0051011 | microtubule minus-end binding | NIN, HAUS4, TUBGCP5, CAMSAP1 | MF | 0.029961 |
| GO:0070063 | RNA polymerase binding | CCNT1, DHX9, STOM, PKN2, ANP32B | MF | 0.013165 |

**Supplementary Table4.** List of differential metabolites in anion mode (A549 vs AH)

| **No.** | **rt** | **mz** | **p.value** | **log2(FC)** | **VIP** | **ID** | **compound.name** | **adduct** | **Formula** |
| --- | --- | --- | --- | --- | --- | --- | --- | --- | --- |
|  |  |  |  | **T1VST2** |  |  |  |  |  |
| 1 | 0.75 | 118.0505 | 0.001109 | -0.7027 | 0.871498 | C00188 | L-Threonine | M-H | C4H9NO3 |
| 2 | 0.76 | 132.0298 | 0.000372 | -0.9406 | 2.17292 | C00049 | L-Aspartic acid | M-H | C4H7NO4 |
| 3 | 0.76 | 242.0794 | 0.000964 | -2.3918 | 2.37846 | C00475 | Cytidine | M-H | C9H13N3O5 |
| 4 | 0.76 | 146.0453 | 0.000633 | -0.6176 | 3.60871 | C00025 | L-Glutamic acid | M-H | C5H9NO4 |
| 5 | 0.85 | 151.0256 | 0.002322 | -0.4815 | 4.19237 | C07599 | Oxypurinol | M-H | C5H4N4O2 |
| 6 | 0.89 | 160.0611 | 0.009592 | -0.7265 | 0.594585 | C01046 | N-methyl-L-glutamic Acid | M-H | C6H11NO4 |
| 7 | 0.9 | 283.0683 | 0.003405 | -0.9171 | 2.33348 | C01762 | Xanthosine | M-H | C10H12N4O6 |
| 8 | 6.68 | 187.097 | 0.022537 | 0.55089 | 0.96337 | C08261 | Azelaic acid | M-H | C9H16O4 |
| 9 | 9.66 | 351.2174 | 0.000451 | 2.59263 | 0.931982 | C00696 | Prostaglandin D2 | M-H | C20H32O5 |
| 10 | 12.4 | 313.2383 | 0.048836 | 0.8812 | 0.553541 | C14828 | 9,10-DHOME | M-H | C18H34O4 |
| 11 | 14.3 | 271.2276 | 0.024475 | -0.5749 | 0.630085 | HMDB0061658 | 3-Hydroxyhexadecanoic acid | M-H | C16H32O3 |
| 12 | 14.7 | 271.2273 | 4.48E-05 | -1.3928 | 1.10001 | HMDB0112192 | 6-Hydroxyhexadecanoic acid | M-H | C16H32O3 |
| 13 | 15.4 | 269.212 | 9.16E-05 | -2.4864 | 1.93958 | C19614 | 16-Oxohexadecanoic acid | M-H | C16H30O3 |
| 14 | 15.4 | 325.1846 | 0.005174 | 0.36137 | 1.12052 | HMDB0014602 | Acitretin | M-H | C21H26O3 |
| 15 | 15.9 | 299.2585 | 7.55E-06 | -1.1905 | 0.977271 | C03195 | xi-10-Hydroxyoctadecanoic acid | M-H | C18H36O3 |
| 16 | 16 | 295.2276 | 0.001329 | -1.7871 | 1.25007 | C19617 | 18-Oxooleate | M-H | C18H32O3 |
| 17 | 16.2 | 283.264 | 0.035861 | -0.5325 | 0.618077 | C01530 | Stearic acid | M-H | C18H36O2 |
| 18 | 16.8 | 271.2274 | 0.002846 | -1.9046 | 1.85561 | C18218 | 16-Hydroxy hexadecanoic acid | M-H | C16H32O3 |
| 19 | 17.3 | 297.2431 | 0.000869 | -2.3115 | 2.09062 | HMDB0031127 | 5-Hexyltetrahydro-2-furanoctanoic acid | M-H | C18H34O3 |
| 20 | 18.1 | 303.2316 | 0.001405 | 0.67134 | 9.60357 | C00219 | Arachidonate | M-H | C20H32O2 |
| 21 | 18.8 | 329.2479 | 0.000626 | 1.18128 | 3.51468 | C16513 | Docosapentaenoic acid (22n-3) | M-H | C22H34O2 |
| 22 | 19.2 | 305.2478 | 0.000378 | 1.96103 | 3.54046 | C03242 | 8,11,14-Eicosatrienoic acid | M-H | C20H34O2 |

**Supplementary Table5.** List of differential metabolite in anion mode (H1299 vs AH)

| **No.** | **rt** | **mz** | **p.value** | **log2(FC)** | **VIP** | **ID** | **compound.name** | **adduct** | **Formula** |
| --- | --- | --- | --- | --- | --- | --- | --- | --- | --- |
| 1 | 0.85 | 151.0256 | 0.030356 | 0.276992 | 6.1402 | C07599 | Oxypurinol | M-H | C5H4N4O2 |
| 2 | 0.86 | 243.0618 | 0.012349 | -0.52795 | 6.80692 | C00299 | Uridine | M-H | C9H12N2O6 |
| 3 | 0.9 | 320.0618 | 0.004608 | -0.92963 | 6.63229 | C20775 | beta-Citryl-L-glutamate | M-H | C11H15NO10 |
| 4 | 13.31 | 271.2277 | 0.035708 | -0.44778 | 0.7401 | HMDB0031057 | 2-Hydroxyhexadecanoic acid | M-H | C16H32O3 |

**Supplementary Table6**. List of differential metabolite in cationic mode (H1299 vs AH)

| No. | rt | mz | p.value | log2(FC) | VIP | ID | compound.name | adduct | Formula |
| --- | --- | --- | --- | --- | --- | --- | --- | --- | --- |
| 1 | 0.75 | 162.1119 | 0.008162 | -0.37068 | 6.63014 | C00318 | L-Carnitine | M+H | C7H15NO3 |
| 2 | 0.78 | 310.1142 | 0.012215 | -0.60373 | 2.00188 | C00270 | N-Acetylneuraminate | M+H | C11H19NO9 |
| 3 | 0.78 | 112.0502 | 0.024329 | 0.846996 | 5.84769 | C00380 | Cytosine | M+H | C4H5N3O |
| 4 | 0.78 | 244.0933 | 0.04085 | 0.715283 | 3.81982 | C00475 | Cytidine | M+H | C9H13N3O5 |
| 5 | 0.81 | 160.1326 | 0.004128 | -0.72236 | 6.26217 | HMDB0000991 | DL-2-Aminooctanoic acid | M+H | C8H17NO2 |
| 6 | 0.86 | 269.0884 | 0.025886 | -0.48715 | 7.25281 | C00294 | Inosine | M+H | C10H12N4O5 |
| 7 | 12.12 | 298.2747 | 0.046162 | 0.319307 | 0.673712 | HMDB13648 | Palmitoleoylethanolamde | M+H | C18H35NO2 |
| 8 | 12.71 | 313.2382 | 0.021456 | -0.76314 | 0.741326 | C07354 | (7S,8S)-DiHODE | M+H | C18H32O4 |
| 9 | 15.57 | 522.3564 | 0.014663 | 0.377971 | 2.13056 | HMDB0010385 | LysoPC(18:1(11Z)/0:0) | M+H | C26H52NO7P |
| 10 | 17.96 | 171.1381 | 0.043399 | 0.665119 | 1.37503 | C18202 | 8-Methylnonenoate | M+H | C10H18O2 |
| 11 | 19.51 | 301.2745 | 0.021056 | 0.498975 | 2.18249 | C03195 | 10-Hydroxystearic acid | M+H | C18H36O3 |

**Supplementary Table7.** List of differential metabolites in cationic mode (A549 vs AH)

| **No.** | **rt** | **mz** | **p.value** | **log2(FC)** | **VIP** | **ID** | **compound.name** | **adduct** | **Formula** |
| --- | --- | --- | --- | --- | --- | --- | --- | --- | --- |
|  |  |  |  | **T1VST2** |  |  |  |  |  |
| 1 | 0.65 | 112.0866 | 0.011711 | 1.672812 | 1.21349 | C00388 | Histamine | M+H | C5H9N3 |
| 2 | 0.67 | 170.0926 | 0.024942 | -0.48113 | 0.48385 | C01152 | 1-Methylhistidine | M+H | C7H11N3O2 |
| 3 | 0.7 | 175.1188 | 0.001 | 1.161049 | 2.41431 | C00062 | L-Arginine | M+H | C6H14N4O2 |
| 4 | 0.75 | 162.1119 | 0.003853 | 0.445489 | 4.13095 | C00318 | L-Carnitine | M+H | C7H15NO3 |
| 5 | 0.76 | 132.0762 | 0.041215 | -0.45085 | 3.59694 | C00300 | Creatine | M+H | C4H9N3O2 |
| 6 | 0.76 | 427.0967 | 0.02331 | -0.92374 | 1.88101 | HMDB0000656 | Cysteineglutathione disulfide | M+H | C13H22N4O8S2 |
| 7 | 0.78 | 112.0502 | 0.000277 | -4.59368 | 5.67367 | C00380 | Cytosine | M+H | C4H5N3O |
| 8 | 0.78 | 244.0933 | 0.000236 | -3.72255 | 4.03145 | C00475 | Cytidine | M+H | C9H13N3O5 |
| 9 | 0.78 | 310.1142 | 4.25E-05 | 1.220495 | 1.99623 | C00270 | N-Acetylneuraminate | M+H | C11H19NO9 |
| 10 | 0.81 | 160.1326 | 0.001883 | 0.986761 | 4.29578 | HMDB0000991 | DL-2-Aminooctanoic acid | M+H | C8H17NO2 |
| 11 | 0.83 | 123.0548 | 0.006778 | -0.74703 | 5.89588 | C00153 | Niacinamide | M+H | C6H6N2O |
| 12 | 0.83 | 137.0448 | 0.002495 | -0.83733 | 15.2182 | C00262 | Hypoxanthine | M+H | C5H4N4O |
| 13 | 0.83 | 284.0996 | 0.00471 | -2.68925 | 3.87177 | C00387 | Guanosine | M+H | C10H13N5O5 |
| 14 | 0.89 | 218.1391 | 2.10E-05 | 1.519893 | 3.2188 | C03017 | Propionylcarnitine | M+H | C10H19NO4 |
| 15 | 0.9 | 220.1182 | 0.007977 | -0.77781 | 1.86852 | C00864 | Pantothenic acid | M+H | C9H17NO5 |
| 16 | 6.66 | 193.0386 | 0.001548 | 0.456918 | 0.37283 | C00679 | 5-Dehydro-4-deoxy-D-glucarate | M+H | C6H8O7 |
| 17 | 10.65 | 583.2577 | 0.028414 | 0.767656 | 0.44081 | C00500 | Biliverdin | M+H | C33H34N4O6 |
| 18 | 11 | 318.2991 | 0.020528 | 0.256567 | 4.01237 | C12144 | Phytosphingosine | M+H | C18H39NO3 |
| 19 | 12.12 | 298.2747 | 0.004703 | 0.489704 | 0.59459 | HMDB13648 | Palmitoleoylethanolamde | M+H | C18H35NO2 |
| 20 | 12.5 | 315.2 | 0.00218 | 0.94139 | 0.29 | C14828 | 9,10-DHOME | M+H | C18H34O4 |
| 21 | 14.81 | 400.3422 | 0.032607 | -0.78234 | 4.84915 | C02990 | L-Palmitoylcarnitine | M+H | C23H45NO4 |
| 22 | 14.93 | 522.3553 | 0.005158 | -0.93695 | 6.06027 | HMDB0002815 | LysoPC(18:1(9Z)) | M+H | C26H52NO7P |
| 23 | 15.26 | 522.3552 | 0.014388 | -0.9331 | 11.2924 | HMDB0061701 | LysoPC(0:0/18:1(9Z)) | M+H | C26H52NO7P |
| 24 | 15.34 | 271.2276 | 0.000448 | -2.77051 | 0.6084 | C19614 | 16-Oxohexadecanoic acid | M+H | C16H30O3 |
| 25 | 15.57 | 522.3564 | 0.001263 | 0.648822 | 1.99151 | HMDB0010385 | LysoPC(18:1(11Z)/0:0) | M+H | C26H52NO7P |
| 26 | 16.23 | 283.2636 | 0.009243 | -0.89812 | 1.02158 | C01712 | Elaidic acid | M+H | C18H34O2 |
| 27 | 16.31 | 287.2223 | 0.030068 | 0.297705 | 0.5152 | C19615 | Hexadecanedioic acid | M+H | C16H30O4 |
| 28 | 16.34 | 524.3711 | 0.003833 | 0.60214 | 2.51052 | C04230 | LysoPC(18:0) | M+H | C26H54NO7P |
| 29 | 16.43 | 227.2008 | 0.00083 | -0.96864 | 0.59311 | HMDB0005051 | Tsuzuic acid | M+H | C14H26O2 |
| 30 | 16.5 | 299.2589 | 0.002694 | 0.287071 | 0.51181 | HMDB34074 | 5-Oxooctadecanoic acid | M+H | C18H34O3 |
| 31 | 16.71 | 524.3702 | 2.96E-05 | 0.892063 | 18.4601 | HMDB0011128 | LysoPC(0:0/18:0) | M+H | C26H54NO7P |
| 32 | 17.03 | 450.32 | 0.038407 | 0.167035 | 0.69879 | C05464 | Deoxycholic acid glycine conjugate | M+H | C26H43NO5 |
| 33 | 17.18 | 299.2592 | 0.006678 | -0.65259 | 0.69418 | HMDB30979 | 9-Oxooctadecanoic acid | M+H | C18H34O3 |
| 34 | 17.27 | 239.2372 | 0.000503 | -1.45684 | 0.8992 | C06123 | Hexadecenal | M+H | C16H30O |
| 35 | 17.48 | 326.3062 | 0.041163 | -0.89186 | 1.52156 | HMDB0002088 | N-Oleoylethanolamine | M+H | C20H39NO2 |
| 36 | 17.85 | 305.2489 | 0.035505 | -0.80048 | 1.36722 | C00219 | Arachidonate | M+H | C20H32O2 |
| 37 | 18.12 | 257.2475 | 0.011041 | -2.0012 | 0.89094 | C00249 | Palmitic acid | M+H | C16H32O2 |
| 38 | 18.13 | 305.2467 | 0.001315 | 0.93108 | 14.9667 | HMDB0002177 | Cis-8,11,14,17-Eicosatetraenoic acid | M+H | C20H32O2 |
| 39 | 18.13 | 331.2847 | 0.008204 | -2.42975 | 5.00982 | HMDB11533 | MG(0:0/16:0/0:0) | M+H | C19H38O4 |
| 40 | 18.42 | 239.237 | 0.00484 | -0.44925 | 1.6497 | HMDB0034181 | 3-Methylcyclopentadecanone | M+H | C16H30O |
| 41 | 18.42 | 257.2484 | 0.028456 | -0.44205 | 0.45924 | HMDB0031068 | Isopalmitic acid | M+H | C16H32O2 |
| 42 | 18.42 | 331.2842 | 0.001778 | -0.46977 | 2.92899 | HMDB0011564 | MG(16:0/0:0/0:0) | M+H | C19H38O4 |
| 43 | 18.59 | 281.248 | 0.000865 | -1.36794 | 2.39553 | C01595 | Linoleic acid | M+H | C18H32O2 |
| 44 | 18.67 | 283.2635 | 0.000456 | -1.2796 | 1.27147 | C00712 | Oleic acid | M+H | C18H34O2 |
| 45 | 19.17 | 307.2632 | 0.001034 | 1.907154 | 4.91531 | C16522 | Dihomo-alpha-linolenic acid | M+H | C20H34O2 |
| 46 | 19.5 | 283.2624 | 0.00154 | -0.96138 | 11.6705 | C08367 | Vaccenic acid | M+H | C18H34O2 |
| 47 | 19.51 | 301.2745 | 0.002645 | -0.79799 | 1.5674 | C03195 | 10-Hydroxystearic acid | M+H | C18H36O3 |
| 48 | 19.74 | 291.2686 | 2.78E-05 | -1.48538 | 3.18188 | C00353 | Geranylgeranyl-PP | M+H | C20H34O |
| 49 | 19.74 | 309.2791 | 7.68E-08 | -1.61758 | 3.21902 | C16525 | Eicosadienoic acid | M+H | C20H36O2 |
| 50 | 20.12 | 585.2719 | 0.045128 | 0.774441 | 1.69061 | C00486 | Bilirubin | M+H | C33H36N4O6 |
| 51 | 20.61 | 401.3421 | 0.000518 | 1.650016 | 3.2193 | HMDB0000501 | 7-Ketocholesterol | M+H | C27H44O2 |

**Supplementary Table8**. List of Abbreviations

| **Abbreviation** | **Full Name** |
| --- | --- |
| NSCLC | Non–small-cell lung carcinomas |
| EVs | Extracellular vesicles |
| MVBs | Multivesicular bodies |
| PUFAs | Polyunsaturated fatty acids |
| mRNA | messenger RNA |
| TME | Tumor microenvironment |
| ROS | Reactive oxygen species |
| ctDNA | Circulating tumor DNA |
| FOXO | Forkhead box O |
| FOXO4 | Forkhead box O4 |
| SOD | Superoxide dismutase |
| DAPI | 4’,6-Diamidino-2-phenylindole |
| SEM | Scanning electron microscope |
| PBS | Phosphate buffered saline |
| DMEM | Dulbecco”s modification of eagle”s medium |
| QC | Quality control |
| MS | Mass spectrometry |
| GEO | Gene expression omnibus |
| GO | Gene ontology |
| BP | Biological process |
| CC | Cellular component |
| MF | Molecular function |
| KEGG | Kyoto encyclopedia of genes and genomes |
| SMILES | Simplified molecular input line entry system |
| PPI | Protein-protein interaction |
| TCGA | the Cancer genome atlas |
| PCR | Polymerase chain reaction |
| RT-qPCR | Reverse transcription quantitative-polymerase chain reaction |
| GAPDH | Glyceraldehyde-3-phosphate dehydrogenase |
| H&E | Hematoxylin-eosin |
| IHC | Immunohistochemistry |
| SDS-PAGE | SDS-Polyacrylamide gel electrophoresis |
| PVDF | Polyvinylidene fluoride |
| GFP | Green fluorescent protein |
| LUAD | Lung adenocarcinoma |
| GFP | Green fluorescent protein |
| SPSS | Statistical product and service solutions |
| AH cells | Co-cultured A549 and H1299 cells |
| TME | Tumor microenvironment |
| TGFβ1 | Transforming growth factor-β1 |
| ROOH | Lipid peroxides |
| RCCs | Reactive carbonyl compounds |
| SEA | Similarity ensemble approach |
| STRING | Search tool for the retrieval of interaction gene/proteins |
| DAVID | Database for annotation, visualization and integrated discovery |
| HPA | the Human protein atlas |
