# Supplementary material for: Downregulation of 4-HNE and FOXO4 collaboratively promotes NSCLC cell migration and tumor growth
Source: Cell Death Dis. 2024 Jul 31;15(7):546. doi: 10.1038/s41419-024-06948-4 (PMC11291900; doi:10.1038/s41419-024-06948-4)
Supplement: Supplementary file 2 — Uncropped western blots [file 41419_2024_6948_MOESM2_ESM.pptx]

## Slide 1
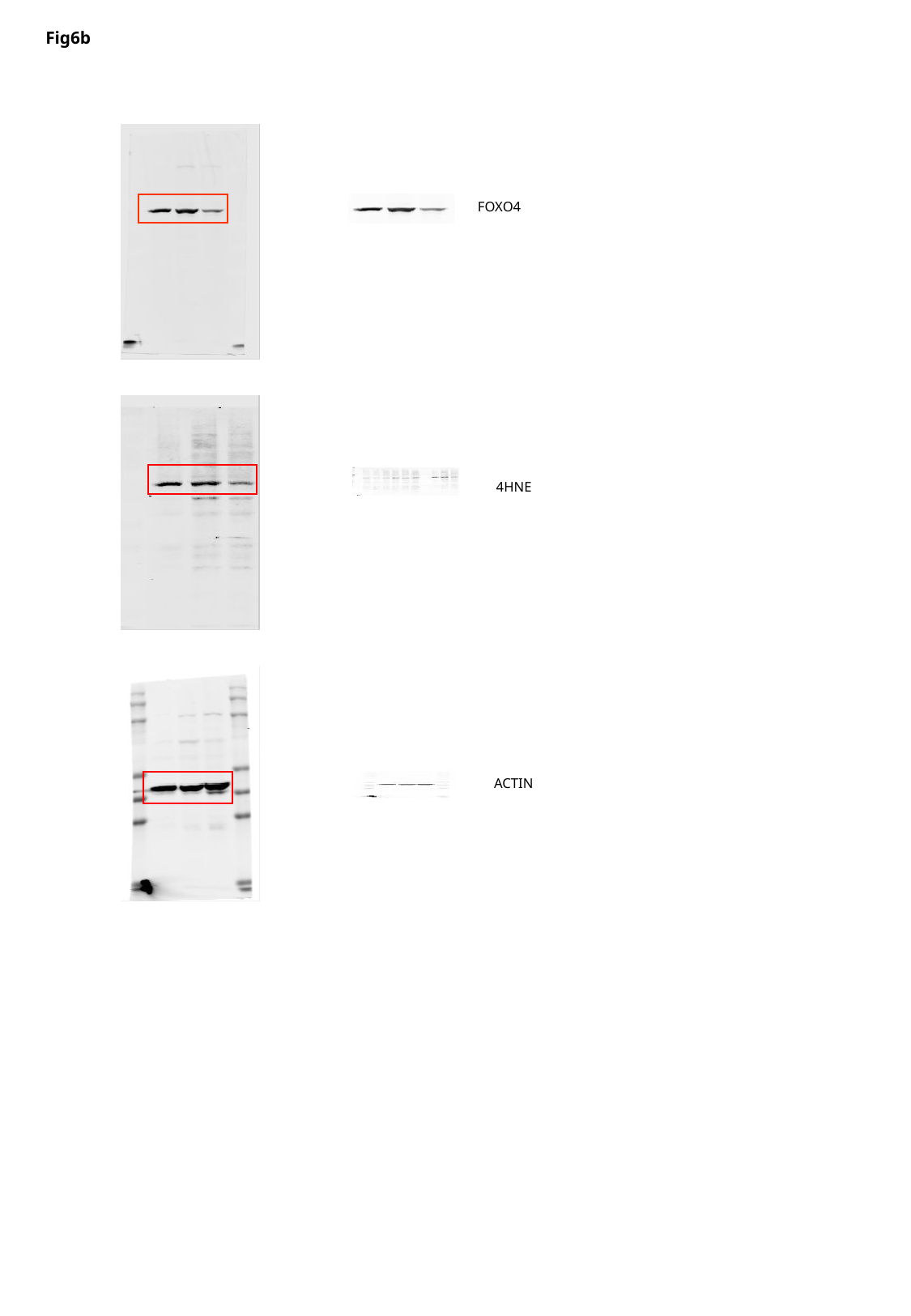

Fig6b
FOXO4
4HNE
ACTIN

## Slide 2
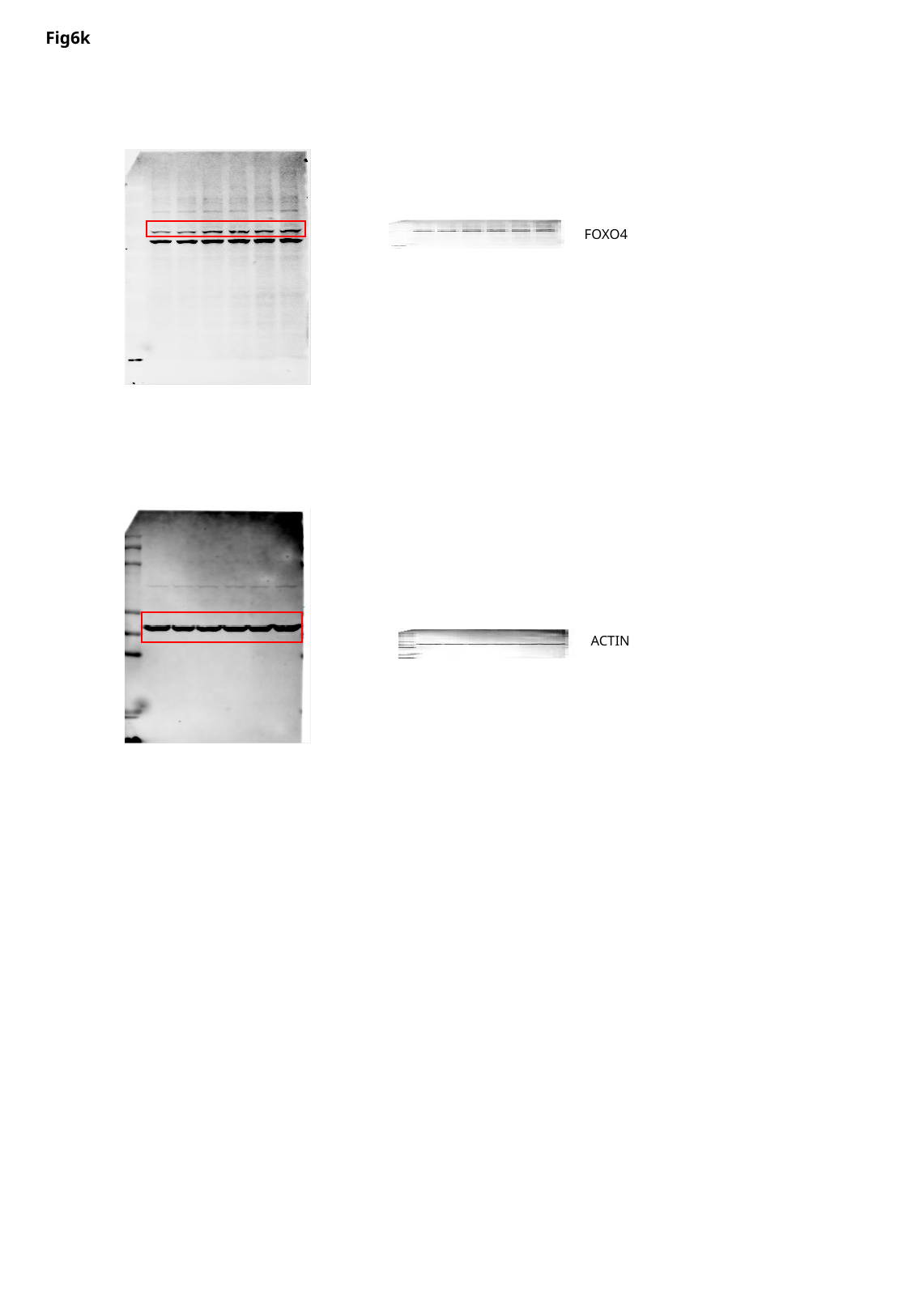

Fig6k
FOXO4
ACTIN

## Slide 3
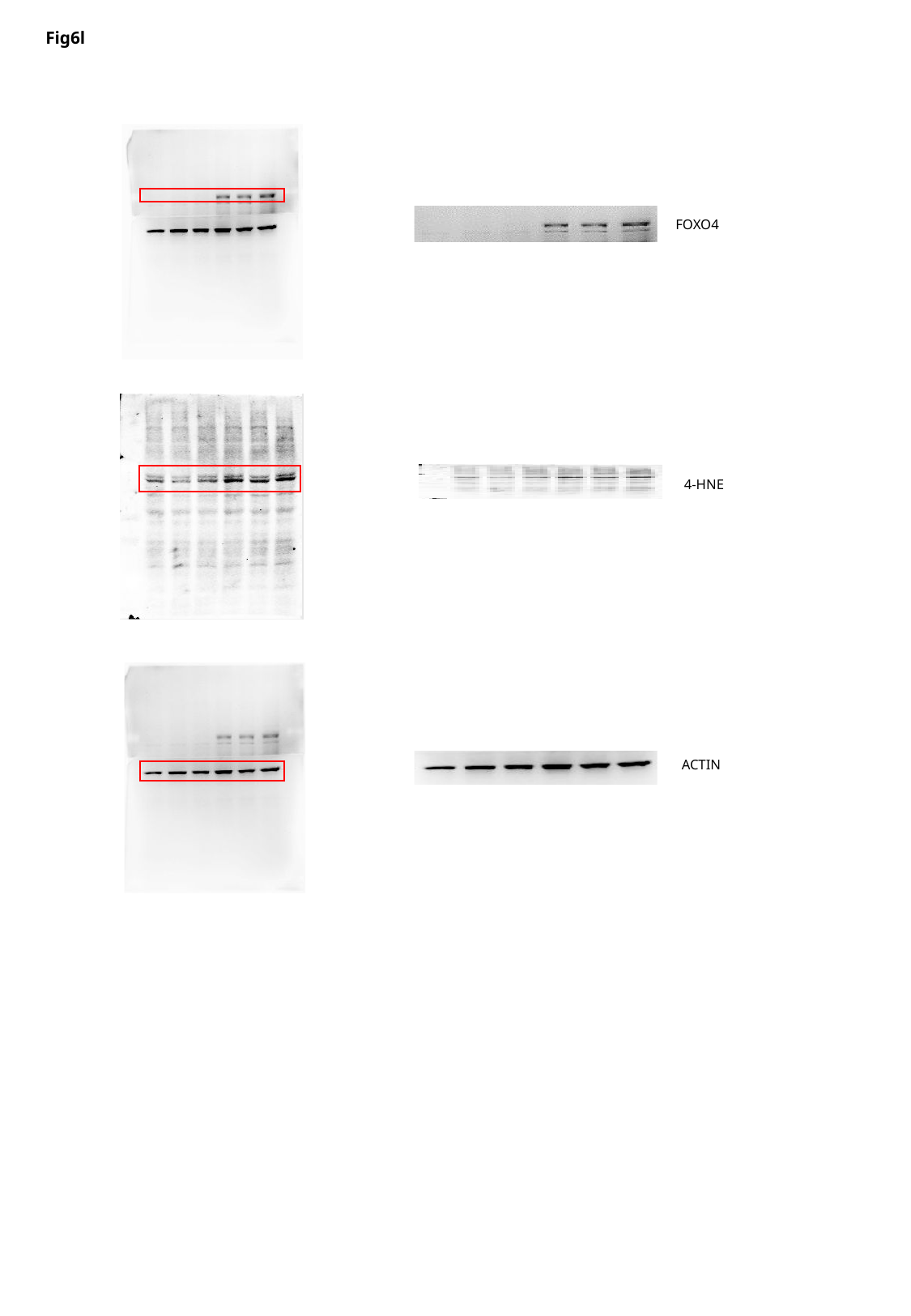

Fig6l
FOXO4
4-HNE
ACTIN

## Slide 4
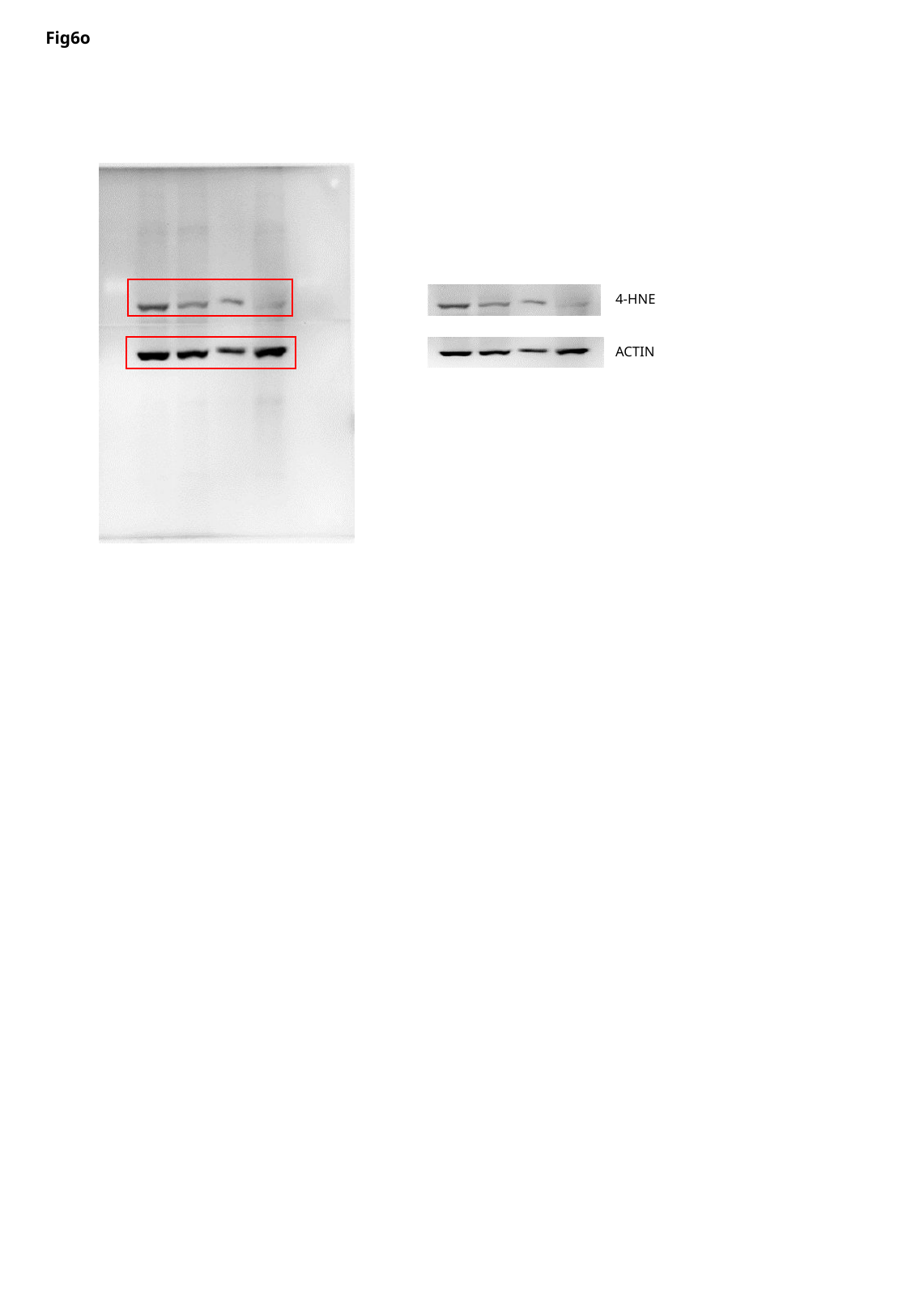

Fig6o
4-HNE
ACTIN

## Slide 5
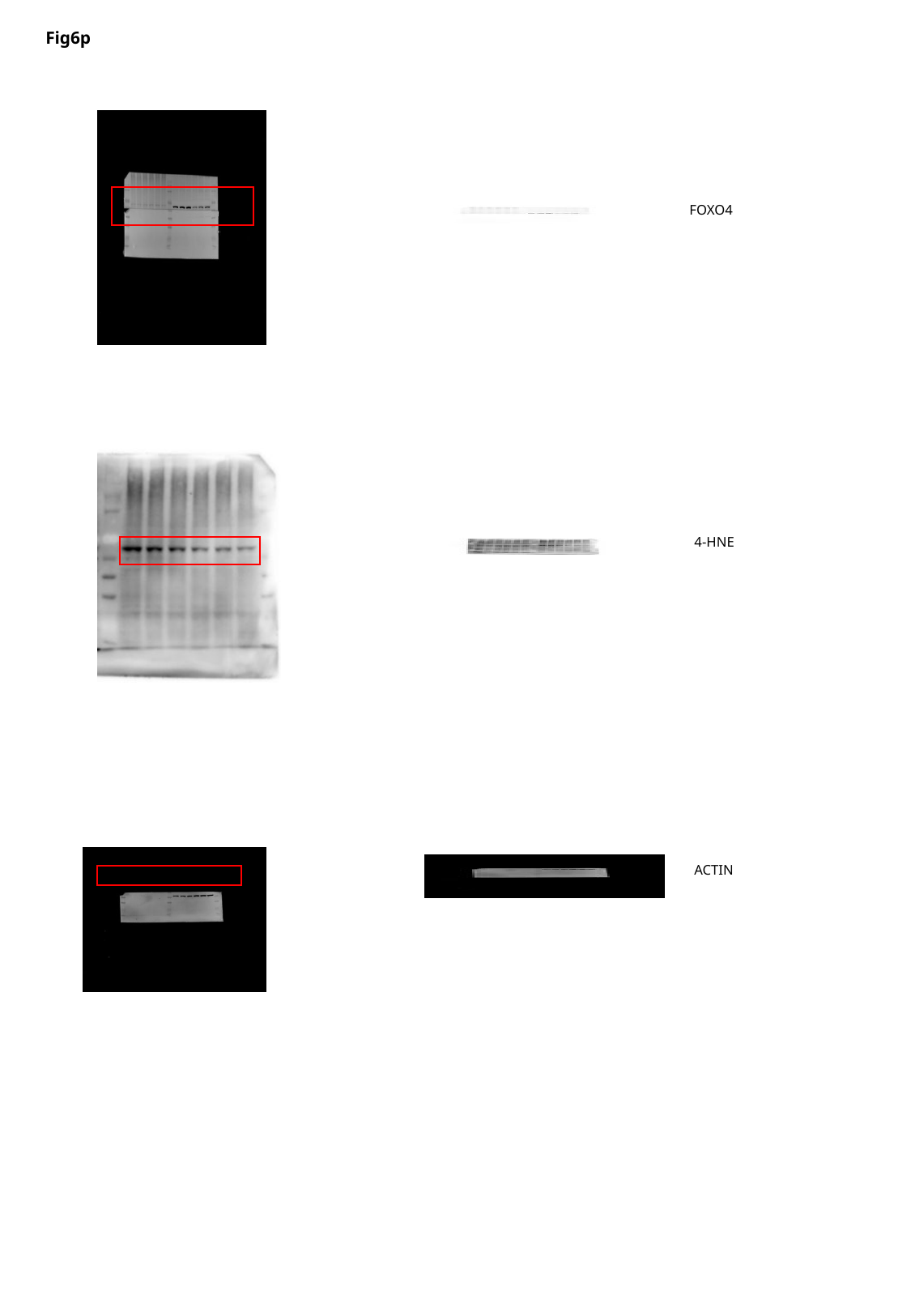

Fig6p
FOXO4
4-HNE
ACTIN
